# A machine learning decision criterion for reducing scan time for hyperspectral neutron computed tomography systems

**DOI:** 10.1038/s41598-024-63931-x

**Published:** 2024-07-02

**Authors:** Shimin Tang, Singanallur V. Venkatakrishnan, Mohammad S. N. Chowdhury, Diyu Yang, Megan Gober, George J. Nelson, Maria Cekanova, Alexandru S. Biris, Gregery T. Buzzard, Charles A. Bouman, Harley D. Skorpenske, Hassina Z. Bilheux

**Affiliations:** 1 Oak Ridge National Laboratory, Neutron Scattering Division, Oak Ridge, 37831 USA; 2 Oak Ridge National Laboratory, Electrification and Energy Infrastructure Division, Oak Ridge, 37831 USA; 3 School of Electrical and Computer Engineering, Purdue University, West Lafayette, 47907 USA; 4 Department of Mathematics, Purdue University, West Lafayette, 47907 USA; 5 University of Alabama in Huntsville, Mechanical and Aerospace Engineering, Huntsville, 35899 USA; 6 College of Veterinary Medicine, University of Tennessee, Knoxville, 37932 USA; 7 Center for Integrative Nanotechnology Sciences, University of Arkansas-Little Rock, Little Rock, 72204 USA; 8 Present Address: Integrity Laboratories, Knoxville, 37932 USA

**Keywords:** Characterization and analytical techniques, Computer science

## Abstract

We present the first machine learning-based autonomous hyperspectral neutron computed tomography experiment performed at the Spallation Neutron Source. Hyperspectral neutron computed tomography allows the characterization of samples by enabling the reconstruction of crystallographic information and elemental/isotopic composition of objects relevant to materials science. High quality reconstructions using traditional algorithms such as the filtered back projection require a high signal-to-noise ratio across a wide wavelength range combined with a large number of projections. This results in scan times of several days to acquire hundreds of hyperspectral projections, during which end users have minimal feedback. To address these challenges, a golden ratio scanning protocol combined with model-based image reconstruction algorithms have been proposed. This novel approach enables high quality real-time reconstructions from streaming experimental data, thus providing feedback to users, while requiring fewer yet a fixed number of projections compared to the filtered back projection method. In this paper, we propose a novel machine learning criterion that can terminate a streaming neutron tomography scan once sufficient information is obtained based on the current set of measurements. Our decision criterion uses a quality score which combines a reference-free image quality metric computed using a pre-trained deep neural network with a metric that measures differences between consecutive reconstructions. The results show that our method can reduce the measurement time by approximately a factor of five compared to a baseline method based on filtered back projection for the samples we studied while automatically terminating the scans.

## Introduction

Since neutrons interact with the nucleus of an atom, they provide a complementary contrast to X-rays which interact with the atom’s electron cloud. Neutron radiography (nR) utilizes this contrast to measure a plethora of objects ranging from materials science, energy materials to plant physiology, engineering, archeology and biology, to name a few^1,2^. These measurements are often performed at research reactor facilities^3–8^. Recently, with the advances of accelerator-based neutron sources and detector technology^9^, wavelength-dependent or hyperspectral neutron radiography (HSnR) and computed tomography (HSnCT) have grown as essential non-destructive techniques that complement reactor-based neutron imaging capabilities^2,10–13^. These novel techniques allow the measurements of crystalline properties in materials using the so-called Bragg edge imaging method^12,14–16^, or the measurement of elements/isotopes in a broad range of samples^17^ using resonance imaging. Hyperspectral imaging is performed by measuring the neutron’s wavelength using the time-of-flight (TOF) method.

Hyperspectral or TOF neutron imaging beamlines have been developed at several neutron facilities around the world, such as the Energy Resolved Neutron Imaging (ERNI)^18^ at Los Alamos National Laboratory in the US, the Imaging and Materials Science and Engineering (IMAT)^19^ at the Rutherford Appleton Laboratory in the UK, and RADEN^20^ at the Japanese Proton Accelerator Research Complex (J-PARC) in Japan. Several other beamlines are also under construction including the Versatile Neutron imaging Instrument (VENUS) at the Spallation Neutron Source (SNS) in the US^21^, ERNI^22^ at the Chinese Spallation Neutron Source (CSNS) in China, and ODIN at the European Spallation Neutron Source (ESS)^23^. HSnCT provides the ability to obtain neutron computed tomography (nCT) reconstructions corresponding to several wavelength bands, each of which is based on a narrow range of neutron wavelengths. Since the measurement is performed simultaneously across a broad range of neutron wavelengths, the data corresponding to a narrow wavelength bin has inherently lower neutron counts than a reactor-based nCT which does not discriminate neutron wavelengths. Therefore one of the caveats of HSnCT is the low inherent signal-to-noise ratio (SNR) in each wavelength bin when using practical acquisition times (tens of minutes to hours), thus rendering strong artifacts when using traditional reconstruction methods^24–26^, such as the filtered back projection (FBP)^27,28^.

At the currently operating neutron imaging facilities, data acquisition and computed tomography (CT) reconstruction are typically performed sequentially. In practice, CT reconstruction is usually done after a pre-decided total number of projections has been acquired (using a fixed set of orientations) typically using baseline analytic reconstruction algorithms such as FBP. At the SNS, an HSnCT projection can be acquired in an hour or more depending on several experimental factors such as neutron flux, sample overall transmission, background at the instrument, detector performance, wavelength and/or pixel binning of the data, etc. Processing and reconstruction of the hyperspectral data may require hours of computing time, which makes real-time experimental decisions challenging. Furthermore, the use of FBP can result in reconstructions with streaks and noise artifacts, yielding a sub-optimal image quality at the end of the experiment unless the exposure time and total number of projections are dramatically increased—which is impractical for HSnCTs (i.e., this would mean increasing the beam time requirements by several days). Moreover, the standard workflow of collecting a fixed number of projections at pre-decided orientations can also lead to an inadequate use of beam time spent collecting projections that do not provide relevant information (i.e., sample too opaque in the direction of the measurement) or do not significantly improve the reconstruction of the sample. Finally, beam time is precious as it is relatively unusual for a research team to access the facility more than once or twice a year. Such special but plausible cases, i.e., long measurement times and high incidence of measurement inadequacies, are the central motivation for real-time monitoring systems designed specifically for neutron imaging facilities, and applicable to other time-consuming CT imaging facilities.

In order to address the challenges of long scan times and limited user feedback, advanced scanning protocols based on the golden ratio method^29^ combined with model-based iterative reconstruction (MBIR) algorithms have recently been proposed^30^ by our team. It was demonstrated that by streaming data to a powerful compute node, and using a fast implementation of MBIR^31^, it was possible to reconstruct CT scans with high image quality while performing an experiment, thereby paving the way to make real-time decision during an experiment. However, even workflows that use MBIR in a streaming manner rely on the acquisition of a fixed number of a priori projections and do not adapt to the specifics of the sample. Therefore, while these workflows help dramatically to reduce scan time compared to the baseline workflows based on FBP, it is challenging to select a fixed sparsity factor that accounts for measurement conditions and the complexity of the samples, potentially leading to more measurements than may be required for a high-quality reconstruction. Furthermore, continuously monitoring an experiment that spans several days and stopping the scan manually based on the visual quality of the reconstructions, even when using the MBIR based workflows, can be impractical.

In this paper, we propose a novel workflow for HSnCT systems for which (1) the measurements are streamed and reconstructed in real time using MBIR, (2) the quality of the resulting reconstruction is assessed based on a machine-learning criterion, and (3) a new sample hyperspectral projection is initiated only if the termination criterion has not been met. One of the key novelties of our approach is the design of the decision criterion based on the 3D reconstructed volume thus far. Specifically, we propose a new quantity, termed reconstruction quality index (rQI), which combines two concepts—a reference-free quality assessment score and a metric that assesses the change in quality between the two latest consecutive 3D volumes. For the rQI, we use a deep neural network (DNN) that is pre-trained from previous nCT scans to assign a user-defined score between 1 (lowest) and 5 (highest) as a proxy for reconstruction quality. In order to evaluate whether the reconstruction quality has improved with the latest measurement, we combine the normalized root mean square error (NRMSE)^32,33^ and structural similarity index measure (SSIM)^34^ between the current and previous reconstructions in a principled manner. Moreover, the reconstruction rQIs from different wavelength bands of interest are computed after each new measurement, and if the averaged rQI from those disparate bands meets a pre-set threshold, the HSnCT scan is terminated. Using hyperspectral experimental data, we demonstrate how our method can result in approximately a factor of 5 reduction in the total number of measurements with a high image quality compared to the baseline protocol which uses a large number of pre-determined views and the FBP reconstruction algorithm. We call our proposed system—HyperCT as in Hyperspectral Computed Tomography in the remainder of this manuscript.

## Methods

### Proposed HyperCT workflow

**Figure 1:**
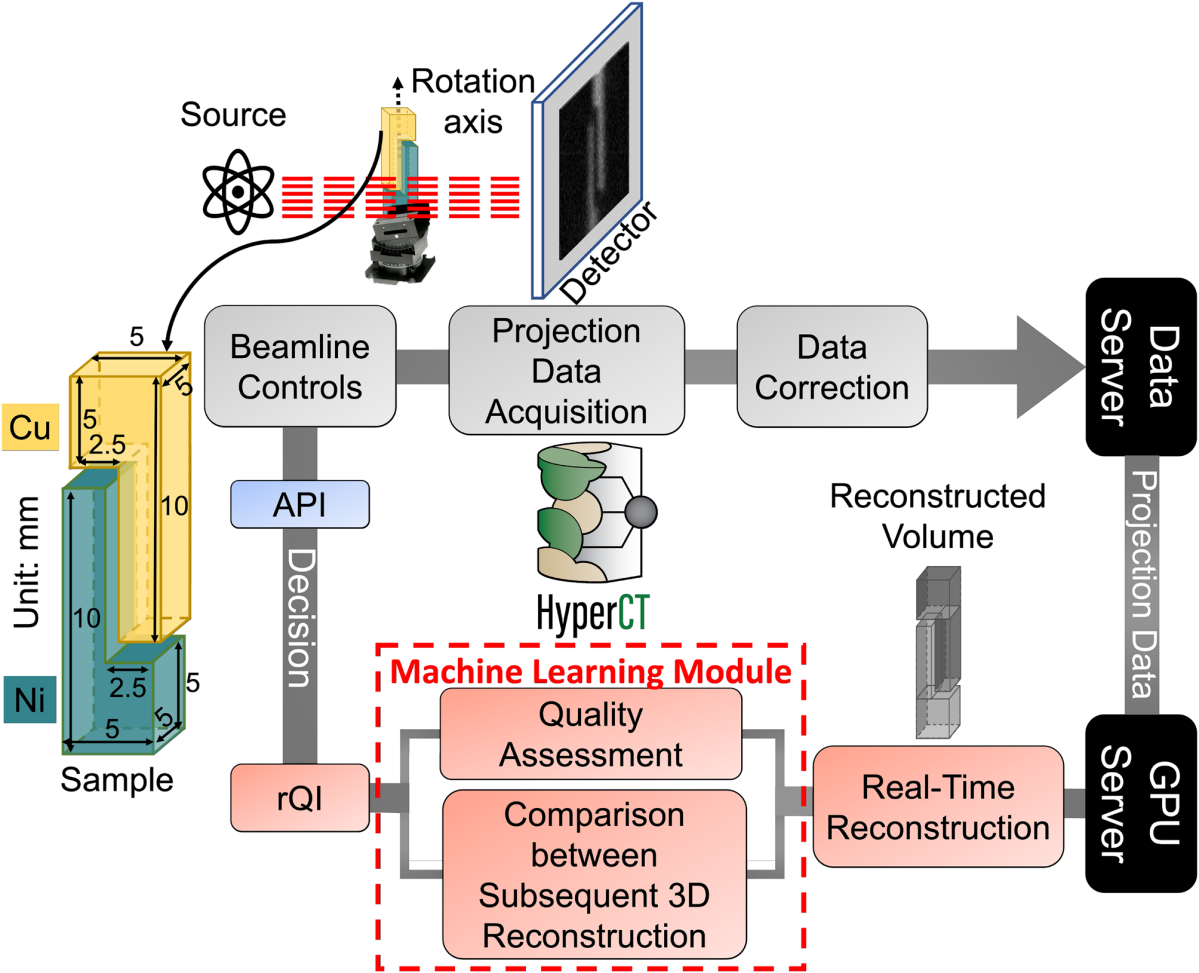
Overall workflow of our streaming HyperCT capability showing the machine learning component of the autonomous loop: The SNS produces neutrons that are either absorbed or scattered by a sample that sits on a rotation stage. A TOF position sensitive 2D detector records neutrons that are transmitted through the sample. As new projections are acquired, they are sent to a GPU server after correction. The reconstruction is performed every few projections (every 3 projections in the case of the data presented in this manuscript). A machine learning reconstruction quality score (rQI) is computed based on the reconstruction generated from all previous projections. Based on this score, the scan either continues or stops. If the scan continues, new projection angles are sent directly to the experimental setup via the beamline controls interface, along with a trigger to resume acquisition.

HyperCT is an autonomous streaming loop that controls the HSnCT measurements and consists of several stages (as shown in Fig. 1): at the SNS, neutrons transmitted through the sample are measured using a microchannel plate (MCP) Timepix (TPX) detector^35^ which is capable of timestamping neutrons as they arrive, thus providing TOF or wavelength/energy information of the neutrons. The measured data is transferred between two servers. After being collected, the hyperspectral projections (constituting of several hundreds of radiographs) are firstly recorded, corrected/reduced, and archived on a core-based data server. A separate server configured with Graphic Processing Units (GPUs) is used for computation and analysis. The GPU server is equipped with four NVIDIA GPUs (A100-PCIE-40GB). The proposed (Python-based) streaming HyperCT system is embedded in the GPU server. The hyperspectral projections are sent to the GPU server after automated data normalization where the super voxel MBIR (svMBIR)^36^ reconstruction is performed. The svMBIR realizes a fast reconstruction of sparse and noisy parallel beam data along with an automated choice of parameters that adapt to the SNR ratio of the measured data. Therefore, the proposed workflow offers an automated real-time reconstruction. The quality score of a single reconstruction is computed and consecutive reconstructions are compared with each other using the rQI, i.e., the reconstructed volume quality will be evaluated based on rQIs. If the automated decision is to continue, HyperCT sends new scanning angles to the rotation stage at the beamline, followed by the command to acquire new projections. The decision command is sent to the beamline via an Application Programming Interface (API), which is the communication protocol between the beamline hardware and the GPU server. In practice, the reconstruction is triggered at a certain frequency and is based on all previously acquired projections. All processed projections, generated reconstructions and evaluation results are archived in real-time on a specific data disk mounted on the GPU server and are accessible to the end user at any time during the experiment. HyperCT allows the end user to choose the projection angles (if preferred), the maximum number of projections, and the rQI threshold value which directly impacts the automated decision to continue or terminate a scan. The threshold value selection is empirical. Based on the rQIs of previous experimental data, the threshold could be set close to the value at which the rQI does not significantly increase with additional projections. Ideally, the choice of threshold should be sample agnostic, but this can be challenging if we only have limited training data. The threshold selection for the experimental measurements used in this paper are described in the implementation section. In summary, HyperCT includes 5 main steps: (1) Projection data acquisition; (2) Automated data correction/reduction followed by archiving on the data server; (3) Real-time CT reconstruction performed on the GPU server; (4) Evaluation of the reconstruction quality on the same server; (5) Decision to continue/stop (GPU server) and new angles submitted to the beamline hardware via an API.

### Design of the stopping criterion

The design of a sample adaptive autonomous streaming HSnCT system that can be trained to automatically continue or terminate a scan requires a stopping criterion which determines if the number of acquired projections is sufficient. To address this challenge, we design a method to assign a quality score to a 3D hyperspectral volume reconstructed using all the measurements made at any point during the scan. Our overall score is a weighted sum of a reference-free quality score designed with a convolutional neural network (CNN) with a score that measures how much the reconstruction changed due to the latest batch of measurements from the streaming system. The first term objectively evaluates if the current 3D reconstruction is of sufficiently high-quality. The second set of terms determines if adding new measurements results in a sufficient change in the 3D reconstruction. The availability of a weighting term allows users to control the fact that if the CNN-based score is not reliable it can be weighted lower compared to the second set of terms. We provide further examples about why both these sets of terms, a subjective image quality and a measure of the difference between successive reconstructions, are important in Appendix A. Next, we present mathematical details for each of these sets of terms followed by a method to combine them into a comprehensive reconstruction quality index, rQI.

#### Using convolutional neural network to evaluate the quality of 3D reconstructions

An accurate 3D reconstruction or tomogram has sufficient spatial resolution when the object edges are sharp, contrast is high, etc., such that the feature(s) of interest can be observed. Reference-free tomogram quality assessments have been proposed in the literature^37,38^ including a CNN based method^39^. In order to associate a notion of user-defined quality score with a 3D reconstructed volume, we propose to use a deep CNN approach. CNN is a classic machine learning tool for imaging applications, such as object detection, recognition, or classification^40–43^. The ability of CNN models to extract low dimensional features has been used for the quality assessment of 2D images^44–46^. Moreover, 3D CNN models have recently been used for the quality assessment of video data^47,48^. Inspired by these efforts and to assess 3D features in a sample, we have implemented a 3D CNN model that can not only learn 2D spatial features of the object but can also capture the relationships between reconstructed slices. This 3D CNN model is used as a reconstruction quality indicator which can assign a numerical score to the reconstructed volume.

**Figure 2:**
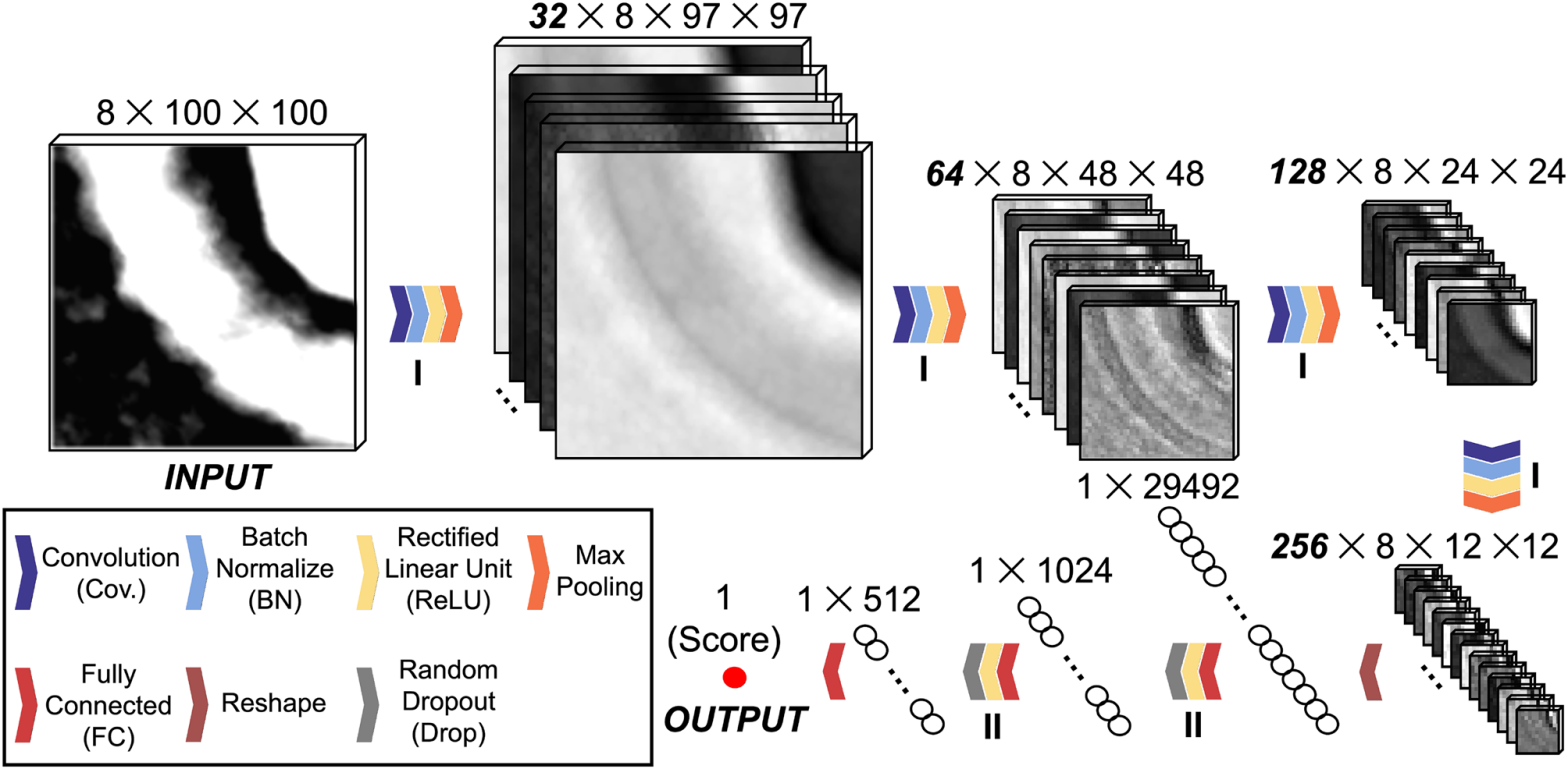
3D CNN network structure (the squares are inner feature examples, and the chevrons are multiple operation layers): Patches from the 3D reconstruction are input to the network and go through 4 convolutional stages I. At each stage, m convolutional filters are first applied. Then the filtered features are normalized, and all negative values are replaced by 0. The features are finally output to the next block after maximum (max) pooling. The convolutional features are in 2D format. Next, they are flattened into 1D vectors and projected to the final quality score via stage II, which includes a fully connected layer, a ReLU layer and a random dropout layer (which randomly drops half of neutrons to contribute to the next layer output).

The proposed 3D CNN quality assessment network structure is shown in Fig. 2. The input to the network is a 3D volume, and the output is a single integer number between 1 and C corresponding to the user-defined image quality of the 3D volume, where C> 1 refers to the highest score. There are four convolutional blocks. Each block consists of a 3D convolution layer, a 3D batch normalization layer, a rectified linear unit (ReLu) layer, and a 3D maximum(max) pooling layer. For the convolution layer, the first two blocks use $3 \times 3 \times 3$ kernels with a stride of 1 and the last two blocks use $2 \times 2 \times 2$ kernels with a stride of 1. We reduced the kernel size in the late phase of the CNN for a better performance because human visualization is more sensitive to smaller spatial regions^47^. The batch normalization is applied to accelerate the training by reducing the internal covariate shift^49^. We use ReLU as the activation in this structure. Furthermore, the 3D max-pooling layer can preserve quality information in a slice by using a $1 \times 2 \times 2$ kernel. The max-pooling is only performed across $2 \times 2$ pixels on slices in the direction of the beam but no pooling between slices is done. The convolutional blocks extract the features of the 3D volume reconstruction at different scales and are followed by three fully connected layers, which flatten the high-dimensional features and transfer them into the final quality score. The depths (number of filters) in each convolutional layer are 32, 64, 128 and 256, respectively, which are chosen to limit the total number of trainable parameters because of the limited availability of training data thus far in its nCT applications. We also utilize a dropout layer to randomly zero some of the features with a probability of 0.5 using samples from a Bernoulli distribution. This is an effective technique for regularization of parameters of the neural networks^50^.

#### Training and testing of the 3D CNN quality score

**Figure 3:**
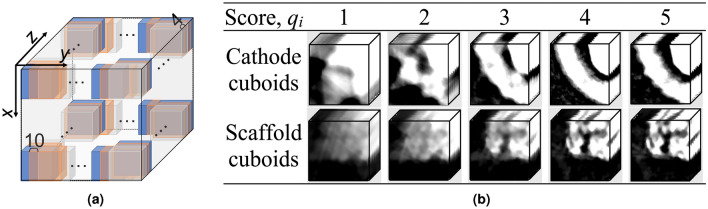
(**a**) Data augmentation scheme: each reconstruction is split into cuboids with a size of $100 \times 100 \times 8$ pixels$^{3}$. The x–y and z strides between two cuboids are 10 and 4 pixels, respectively; (**b**) Illustration of the quality score, qi, attributed to the training data: small cuboids are used to train the quality score evaluator. A few cuboids corresponding to the NMC-cathode (top row) the polymer scaffold (bottom row) reconstructions, respectively. These cuboids are given an integer quality score value between 1 and 5 (where 1 represents a low-quality score and 5 represents the highest).

The proposed model can be trained using previous reconstructions with user-assigned quality scores. Due to the limited amount of hyperspectral data available at the SNS (and in general in the scientific community), we applied a data augmentation scheme to train the deep 3D CNN model using two diametrically opposite samples, i.e., an energy material (cathode) and a bioengineered material (scaffold). The samples are described below. Data augmentation is a technique that artificially increases the training set by creating modified copies of a dataset using existing data. Our general training strategy to generate reconstructions of varying quality is to use previously collected datasets (using a large number of projections), sub-sample the projection data, obtain a sparse-view reconstruction using svMBIR and assign it a quality score. The model is trained using small cuboids (sub-sampling voxels from the reconstruction) instead of full-size reconstructions. Each cuboid can be treated as a small reconstruction with the same score as the original full-size reconstruction. Figure 3a displays how we performed the sub-sampling for data augmentation by using splitting stride sets to 10 and 4 in the x–y plane and along the z-axis, respectively. All reconstructions are split into small cuboids with a size of $100 \times 100 \times 8$ pixels$^{3}$.

Our model is trained using a sub-selection of the number of projections such that different quality reconstructions are obtained. For this research 5, 10, 20, 45, and 60 projections were used. Therefore, a total of 10 reconstructions (5 from each sample) is used for training. Additionally, a quality score is defined between 1 to 5 for each reconstruction, where 1 indicates the poorest quality and 5 the highest. Figure 3b shows examples of cuboids and their labels, i.e., scores, $q_i$, for the cathode and scaffold, respectively.

The performance testing of the quality score evaluator is assessed based on the accuracy of the predicted score of the training data cuboids. In this case, there are 107,760 cuboids created evenly, and 80% are randomly selected as training data while the remainder of the 21,552 cuboids are utilized as test samples. The selected testing cuboids were not used for the training. Moreover, the quality score of the cuboid-based reconstructions is different from a complete 3D reconstructed volume at the time of deployment. The full-size reconstruction is firstly equally split into non-overlapping cuboids with a size of $100 \times 100 \times 8$ pixels${^3}$. To avoid testing the models using the same cuboid sampling pattern used for the training phase, the stride size has a different value from the training dataset. Then, the cuboid scores, $q_i$, are transformed by the trained CNN model. Lastly, the final quality score, $\bar{q_{i}}$ of the corresponding reconstruction, is the averaged quality scores of the cuboids.

#### Improvement evaluation of consecutive 3D reconstructions

Beside the quality of a single reconstruction, the improvement between sequential reconstructions is also an essential evaluation of a streaming system. In this paper, we measure the quality improvement between two successive reconstructions by evaluating the differences between their respective NRMSE and SSIM.

Next, we define the notation that describes our improvement evaluation. Let $x_{kn}$ be the current $k^{th}$ streaming reconstruction from kn projections using svMBIR, where *n*, an integer, is the evaluation frequency for which the reconstructions are triggered and *k* is the incremental number given to each reconstruction. We normalize the root mean square error (RMSE) between $x_{kn}$ and $x_{(k-1)n}$ by the Euclidean norm of the previous $(k-1)^{th}$ reconstruction $x_{(k-1)n}$. Thus, the NRMSE between $x_{kn}$ and $x_{(k-1)n}$ is defined as Eq. (1):

1 $$\begin{aligned} NRMSE_{k;k-1}=\frac{\left\| x_{(k-1)n}-x_{kn}\right\| }{\left\| x_{(k-1)n} \right\| } \end{aligned}$$

where $\left\| (\cdot )\right\|$ refers to the $l_2$ norm. In addition to the NRMSE, we also use the SSIM between subsequent reconstructions because it better accounts for texture (i.e., features) of the image, and is not sensitive to the constant shifts of the pixel value of the image^34^. We define the SSIM between $x_{kn}$ and $x_{(k-1)n}$ as $SSIM_{k;k-1}$.

As the number of projections increases, the difference between two reconstructions should decrease and gradually converge to zero. Conversely, the structural similarity should increase as more projections are added. In general, when the scan covers enough angles, the changes in the reconstruction are minimal. The resulting SSIM is a decimal value between 0 and 1, where 1 indicates perfect similarity, 0 indicates no similarity. However, the NRMSE value range varies among the different samples.

To make all metrics correspond to the same range (so they can be later combined in a single metric, rQI), we assume the first NRMSE obtained during streaming is the largest and denote it as $NRMS_{2;1}$. We save $NRMS_{2;1}$ as the reference, i.e. $NRMS_{2;1}$ = $NRMS_{ref}$. Then, the following NRMSE values are compared to this reference to generate a NRMSE index, NI, as defined in equation (2). Thus, NI values are between 0 and 1, similarly to SSIM. Assuming $NI_{k;k-1}$ is the NI between $x_{kn}$ and $x_{(k-1)n}$, then $NI_{2;1}$=0 and $NI_{k;k-1}$ converges to 1 as *k* increases:

2 $$\begin{aligned} NI_{k;k-1}=1-\frac{NRMSE_{k;k-1}}{NRMSE_{ref}} \end{aligned}$$

Finally, we use the average of SSIM and NI to represent the improvement between subsequent reconstructions which we refer to as the change index (CI) between two consecutive reconstructions. $CI_{k;k-1}$ refers to the primary CI between reconstructions $x_{kn}$ and $x_{(k-1)n}$ and is defined as Eq. (3), where *C* ($C>0$) is defined as a constant to scale SSIM and NI to the same range as the output of 3DCNN.

3 $$\begin{aligned} CI_{k;k-1} = C \cdot SSIM_{k;k-1} + C \cdot NI_{k;k-1} \end{aligned}$$

#### Comprehensive reconstruction quality index (rQI)

We set $\bar{q}_k$ to be the quality score for reconstruction $x_{kn}$ from kn projections using svMBIR. It is an output of the deep learning network. Since a change of the quality score reflects a reconstruction improvement, Eq. (4) defines the difference index (DI) which represents the quantified changes between consecutive reconstructions $x_{kn}$ and $x_{(k-1)n}$. Therefore, DI contains two terms: the scaled NI and the quality score difference corrector which is a weighted difference between consecutive quality scores $\bar{q}_k$ and $\bar{q}_{k-1}$:

4 $$\begin{aligned} DI_{k;k-1} = C \cdot NI_{k;k-1} + \gamma \cdot \left( \bar{q}_k-\bar{q}_{k-1}\right) \end{aligned}$$

where $\gamma$ ($\gamma >0$) is defined as a scalar weight controlling the influence of the score difference corrector. We can use DI to represent improvement between consecutive reconstructions instead of NI itself. Then CI can be rewritten as $CI_{k;k-1} = C \cdot SSIM_{k;k-1} + DI_{k;k-1}$. The overall averaged CI (ACI) becomes:

5 $$\begin{aligned} ACI_{k;k-1}=\frac{C \cdot SSIM_{k;k-1} + DI_{k;k-1}}{2} \end{aligned}$$

Finally, rQI is the weighted average of the sum of the reconstruction quality score and subsequent index. For the reconstruction $x_k$, rQI is defined as:

6 $$\begin{aligned} rQI_k= \alpha \cdot \bar{q}_k + (1- \alpha )\cdot ACI_{k;k-1} \end{aligned}$$

where $\alpha$ is a scalar between 0 and 1 that controls the importance of the quality score and improvement index in the evaluation. Furthermore, multiple $rQI_k^{\Delta \lambda _i}$ are computed in real time for reconstructions at various wavelength bands, $\Delta \lambda _i$, and averaged to get a comprehensive quality score. The final averaged $\overline{rQI}_k^{\Delta \lambda _i}$ is the stopping criterion of the newly developed HyperCT.

### Implementation of HyperCT

#### Sample and dataset descriptions

**Figure 4:**
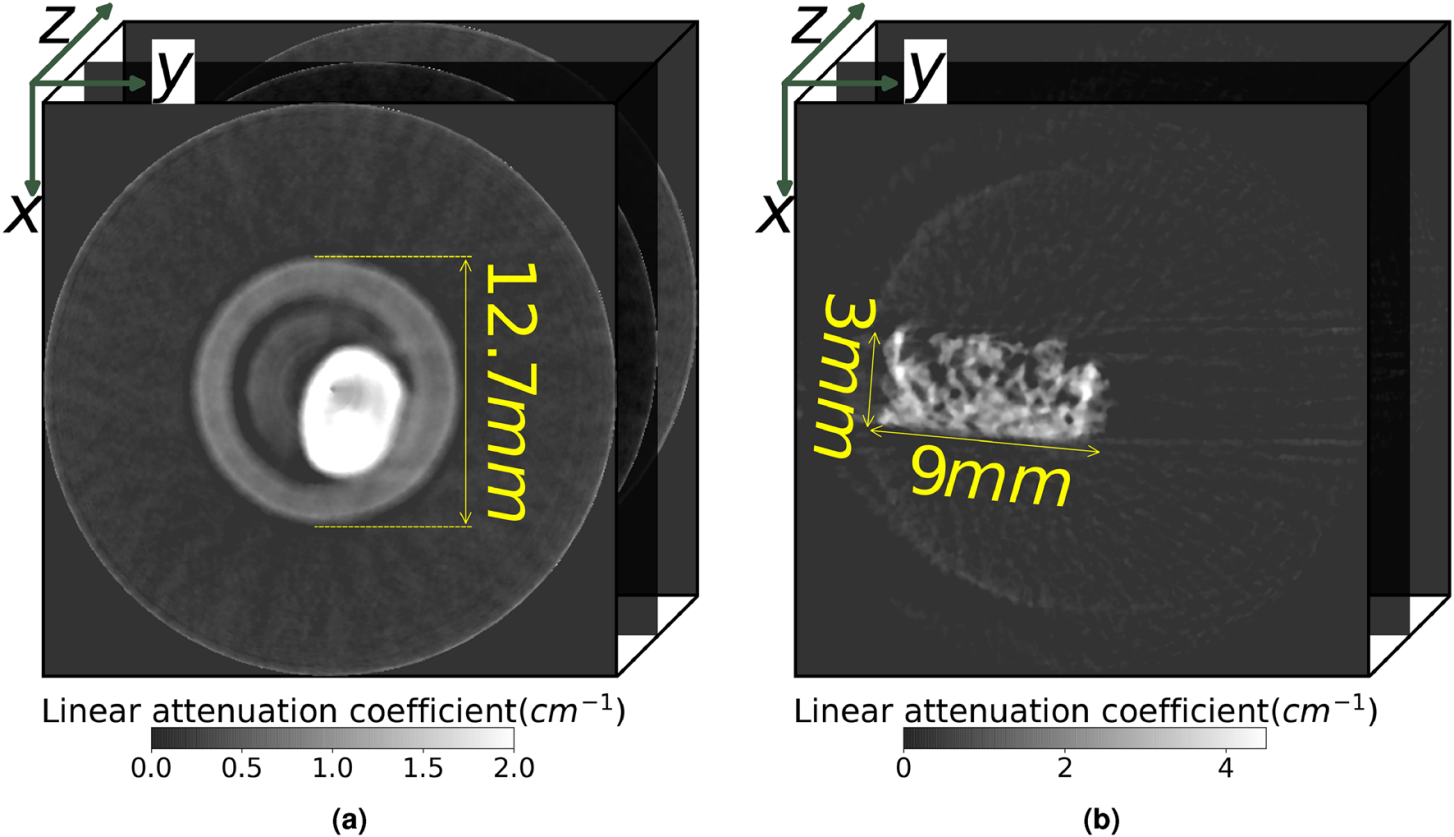
Attenuation-based reconstructed slices in the x-y plane used to train our CNN method; the z-axis corresponds to the sample height: reconstructed slices of (**a**) the NMC cathode and (**b**) the polymer scaffold, respectively. The NMC cathodes are inserted into a Teflon cylinder with a 12.7 mm diameter. The scaffold’s length and width are 9 and 3 mm, respectively.

We utilized two datasets to train the proposed rQI. These two data sets were satisfactory in terms of signal-to-noise ratio (SNR) and overall number of acquired projections. One dataset consists of reconstructions from the measurement of a lithium-ion battery cathode sample at Spallation Neutron Source (SNS) SNAP beamline^51^. Another dataset corresponds to the reconstructions of a polymer-based scaffold which was measured at the High Flux Isotope Reactor (HFIR) Multimodal Advanced Radiography Station (MARS)^52^. The cathodes were fabricated with Li $(Ni_{0.8}Mn_{0.1}Co_{0.1})O_2$ (NMC811) from MSE Supplies LLC as the active material (AM) in an 80:10:10 wt% dry mixture of AM:Carbon Black (MSE Supplies LLC):Polyvinylidene fluoride (PVDF) (Sigma Aldrich) (see Appendix B for more information about the cathode samples). The cathode sample was measured using a microchannel plate (MCP) Timepix detector with a field-of-view of $28 \times 28 mm^2$ developed by A. Tremsin at U.C. Berkeley^35^. The collimation ratio at SNAP was L/D $\sim$ 245, where L = 7.6 m is the distance from the pinhole of diameter, D = 31 mm, and the MCP detector. Neutrons were transported from the pinhole to the detector using helium-filled flight tubes to minimize the loss of neutrons due to air scatter. The detector array is $512 \times 512$, with an effective pixel size of $55 \times 55 \mu m^{2}$. The scaffold sample is made of polyurethane and demineralized de-acellularized bone particles (see Appendix B for more information about the scaffold sample). The scaffold was held in place at MARS using aluminum foil. For these measurements, the L/D ratio was approximately 800 (= 6.559 m and D = 8.2 mm). The large ANDOR DW936 charge coupled device (CCD) with a pixel area of $2048 \times 2048$ and an effective pixel size of $29 \times 29 \mu m^{2}$ was used to measure the scaffold sample. We used the same number of projections for each sample (60 projections) so that not to introduce a new variable to the training. Each reconstruction produces a 3D volume where a series of slices (x–y plane) are stacked along the z-axis. The NMC811 cathode and scaffold volume sizes are $512 \times 512 \times 200$ and $600 \times 600 \times 160$ pixels$^{3}$, respectively. The ideal number of projection measurements^27^ based on the footprint of the sample on the detector are about 250 and 200 scans respectively. Figure 4a and b displays reconstructed attenuation slices from the cathode and the scaffold, respectively, used for the training. A few selected reconstructed slices with the highest attainable image quality (i.e., using both the total number of projections for the reconstruction and svMBIR) are shown along the sample height or z-axis.

#### Implementation of HyperCT using a simulated experiment

We firstly implemented the software of the proposed HyperCT using a simulated experiment. We simulated the neutron imaging CT procedure by fetching the existing scanned projections acquired using the golden ratio method^53^. The frequency at which the quality indices are calculated is set to 5, which means a real-time reconstruction is generated every 5 projections. HyperCT is triggered when the reconstruction is completed. Then, the quality score $\bar{q}_k^{CNN}$ of this full-size reconstruction is evaluated by the proposed 3D CNN model with n = 5. Each full-size reconstruction is split into small cuboids with a size of $100 \times 100 \times 8$ pixels$^{3}$. The $ACI_{k;k-1}$ is computed according to Eq. (5) with C = 5, where C is the highest score of the CNN training dataset that scales the SSIM to values between 1 and 5. The 3D CNN $k^{th}$ rQIs ($rQI_k^{CNN}$) are generated based on Eq. (6) (with $\alpha$=0.6). The value of $\alpha$ is empirical. We weighted quality score $\bar{q}_k^{CNN}$ larger than $ACI_{k;k-1}$ to increase the influence of the CNN based quality score.

In the model training stage, we assigned 5 quality scores from 1 to 5 to reconstructions generated by 5, 10, 20, 45, and 60 projections. Since there is no quality score based on ground-truth reconstructions generated by different numbers of projections, a 3rd degree polynomial regression $q_k^{GT}= \sum _{i=0}^{3}\beta _i(k \cdot n)^i$ was applied for each dataset and correlated to quality scores, where $\beta _i$ is the weight for each polynomial term. With this function, we estimated the ideal/ground-truth score of a reconstruction generated by kn projections even though we did not set it during training. The rQIs ground truth ($rQI_k^{GT}$) is computed by plugging ground truth quality scores, $q_k^{GT}$, and $ACI_{k;k-1}$ , into Eq. (6). By plugging $\bar{q}_k$ of the reconstruction into the simulated experiment, we can generate the simulated $rQI_k$. We also compute the root mean squared error (RMSE) to illustrate the overall difference between computed and ground truth values. The RMSE between the model-generated $rQI_k$ and the ground truth $rQI_k^{GT}$ is defined as:

7 $$\begin{aligned} RMSE=\sqrt{\frac{1}{K}\sum _{k=2}^{K}\left( rQI_k-rQI_k^{GT}\right) ^2} \end{aligned}$$

where *K* is the total number of reconstructions. A representative reconstructed slice is displayed for each sample reconstruction on Fig. 4.

#### Implementation of HyperCT using real-time experimental datasets

Finally, we implemented both the HyperCT hardware and software at the SNS SNAP beamline. The sample consisted of two common elements used in materials science: 2.738 g of nickel (Ni) and 2.337g copper (Cu) powders, respectively. Each powder is poured in an aluminum (Al) holder with dimensions indicated in Fig. 1. The detector array is $512 \times 512$ with an effective pixel size of $55 \times 55 \mu m^{2}$ and the ideal number of projections^27^ for a high-quality reconstruction using the FBP-based workflow for this sample is about 150—which at the rate of about 2 hours per projection is not practical to measure due to the limited availability of beamtime. The sample was mounted on a double stack goniometer purchased from PI^54^. This allowed the sample rotation axis to be tilted and thus provide complementary CT scans with different SNRs since the neutron path through the sample differed from the original scan. Three experiments were therefore performed with the Ni-Cu sample: Experiment I with the sample mounted vertically, Experiments II and III with the sample’s vertical axis tilted by 8$^{\circ }$ and 15$^{\circ }$, respectively. These orientations were chosen to further develop a novel reconstruction algorithm for future optimization of scans using multiple sample tilts (not discussed in this manuscript). However, for the purpose of this paper, we treated each experiment as an individual measurement that we used to test our autonomous stopping criteria algorithm. For these experiments, rQI is computed in real time such that autonomous decisions, i.e., comparison of consecutive reconstructions followed by the decision to stop or continue the scan, are made without human intervention. The neutron wavelengths varied from 1.40 to 4.54 Å, with individual wavelength bands of approximately $2.2 \times 10^{-3}$ Å for each projection, thus creating 1426 radiographs per projection angle. The previously mentioned MCP detector was also used for these measurements. An evaluation frequency n = 3 was chosen for the experiments. The rQI was computed in real-time at n = 2 (i.e., after 2 $\times$ 3 = 6 projections) since a baseline reconstruction is initially required. We selected a stopping value $rQI^{exp}$of 3.88 based on the averaged simulated $rQI_5^{CNN}$, i.e. with 25 hyperspectral projections in total (i.e., a 50 hour experimental time), for the NMC cathode and scaffold. The selection of the rQI value of 3.88 is discussed in the “Results” section.

## Results

The novel HyperCT workflow (see illustration in Fig. 1) is the main contribution of this research. HyperCT uses a 3D CNN model to convert the abstract CT reconstruction quality into a numerical index, and simultaneously considers the consecutive reconstruction changes to generate a quantifiable rQI. HyperCT uses rQI as a criterion to conduct high-efficiency autonomous HSnCT experiment.

### Optimization of the rQI threshold (i.e., stopping criterion) based on simulated workflows (using experimental data)

**Figure 5:**
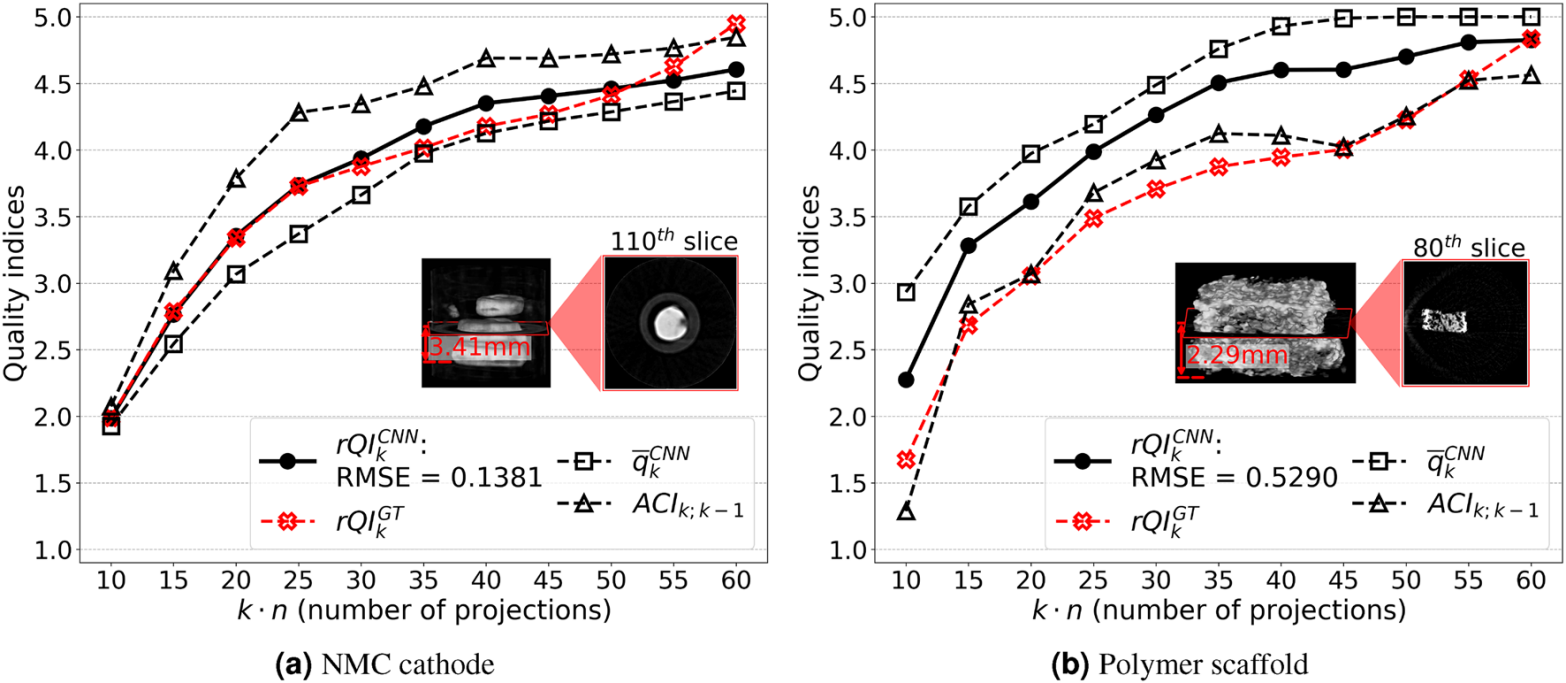
Quality indices ($rQI_{k;k-1}$, $q_k$, and $ACI_{k; k-1}$) as a function of the number of projections used for reconstruction during the simulated experiment. Solid lines represent rQIs, and dashed lines are quality scores, qk, and subsequent indices, ACI. The red crosses correspond to the (user-defined) ground truth $rQI^{GT}$s. Indices are computed using the 3DCNN model for the (**a**) NMC battery electrode and (**b**) polymer scaffold. An example slice from the corresponding reconstruction is also displayed in the plot.

**Figure 6:**
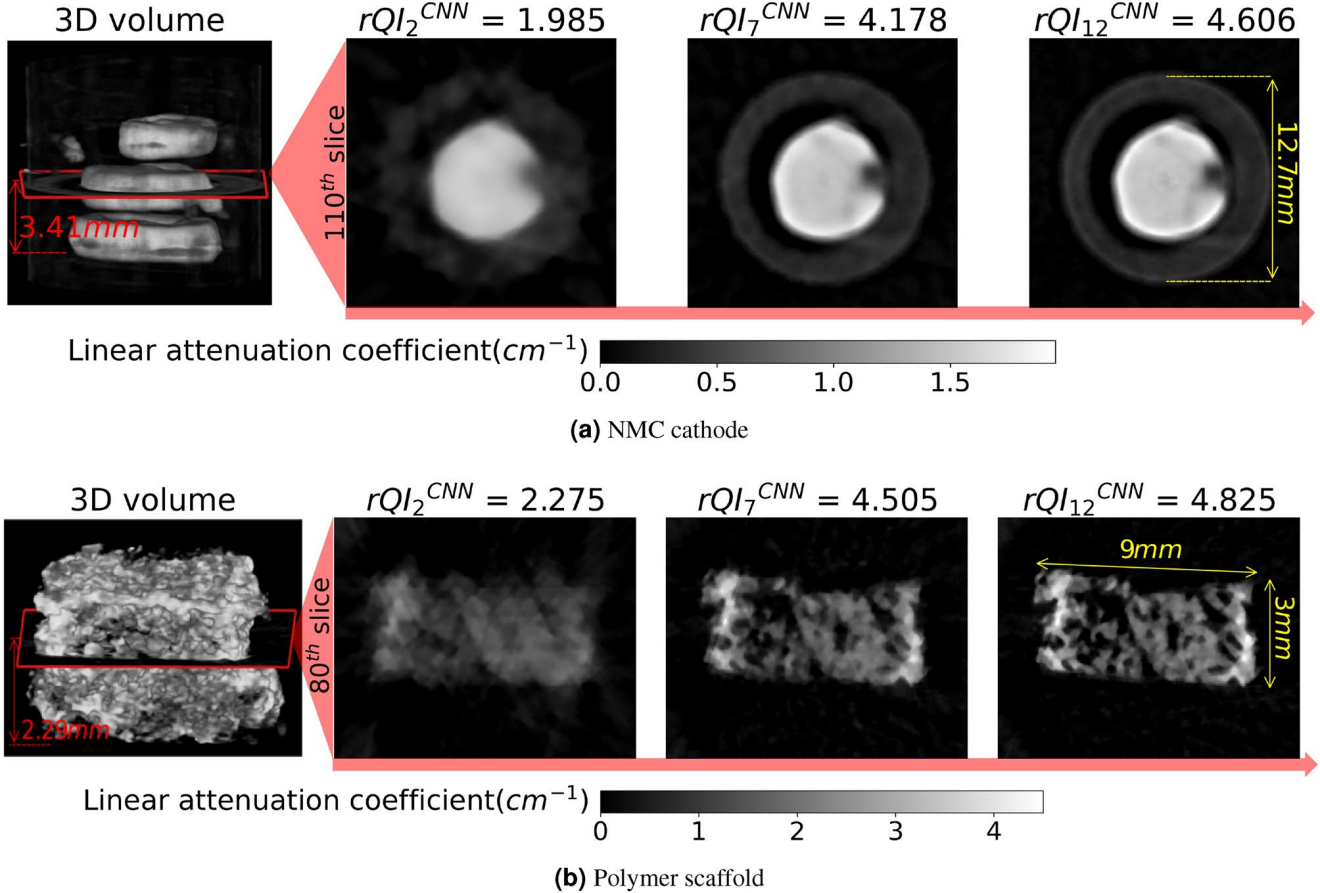
Representative reconstructed slices obtained from the simulated streaming CT for (**a**) the NMC cathode and (**b**) the scaffold samples, respectively: the corresponding estimated rQI of the entire reconstructed object is displayed above each slice. As anticipated, rQI values and slice image quality increase as more projections are utilized for the reconstruction of each object. Slices are extracted from an elevation of 3.41 and 2.29 mm from the bottom of the battery and scaffold samples, respectively.

Figure 5 displays the quality indices, i.e., $\bar{q}_k^{CNN}$, $ACI_{k;k-1}$ and $rQI_k$ for the corresponding reconstruction, *k*, as a function of the projections number, *kn*. These indices are compared to the ground truth results. (We also included details about the performance evaluation in generating $\bar{q}_k$ in Appendix C.) Once $rQI_5^{CNN}$ goes over 3.5, adding projections to the reconstruction only increases the *rQI* value by 1.5 while requiring 20 projections more. In first approximation, changes in the reconstructed data after $x_{25}$ are almost indistinguishable by human eye inspection, as illustrated in Fig. 6 (results discussed below). Therefore, we selected an rQI of 3.88 as the stopping threshold value. Specifically, this value is close to the mid-point of the $rQI_5^{CNN}$ values (i.e. the score when we have about 25 projections and sufficiently high visual quality) for each of the simulated datasets. We note that, the chosen threshold is heuristic and we foresee designing better ways to select the threshold based on a more extensive and representative training data set in future work. Moreover, the cathode *rQI* is in good agreement with $rQI^{GT}$, while the scaffold *rQI* exhibits larger deviations from its respective ground truth. The scaffold RMSE value is approximate 4 times higher than that of the cathode. The indices $\bar{q}_k^{CNN}$ and $ACI_{k;k-1}$ also increase as a function of the number of projections used to reconstruct the data. For the cathode (Fig. 5a), the $\bar{q}_k^{CNN}$ values are lower than the corresponding ACI ones. The opposite behavior is observed for the scaffold (Fig. 5b).

Figure 6 displays the 3D reconstructed sample volume, along with a representative reconstructed slice at a 3.41 and a 2.29 mm elevation from the bottom of the battery and scaffold samples, respectively. The corresponding $rQI_k^{CNN}$ of the entire object is shown above each slice. As illustrated in Fig. 6, the image quality improves with more projections, artifacts decrease, and the rQI value increases concomitantly for both samples. For the battery sample, the improvement of the image quality of the reconstructed slice between 35 ($rQI_7$) and 60 projections ($rQI_{12}$) is visually insignificant which is in agreement with a minimum change in the rQI value. However, for the scaffold sample, the image quality visually improves while the rQI value does not increase significantly. This is due to the bias in training the 3D CNN and the heuristic assignment of quality score values, $\bar{q}_k$.

### Performance of rQI values during real-time experimental measurements

We implemented and tested HyperCT (both hardware and software algorithms) during three experiments at the SNS SNAP beamline using the Nickel-Copper sample displayed in Fig. 1: Experiment I for which the sample was mounted vertically, Experiments II and III with the sample tilted by 8$^{\circ }$ and 15$^{\circ }$, respectively. Once the preset threshold value is reached, the autonomous experiment will stop without human intervention. HyperCT computes a single reconstruction quality score using our 3D CNN model, along with the corresponding average change indices during the measurement. Specifically, *rQIs* ($rQI_k^{\Delta \lambda _1}$ and $rQI_k^{\Delta \lambda _2}$) from multiple neutron wavelength bandwidths, where $\Delta \lambda _1$ = 2.1508 +/− 0.0415Å and $\Delta \lambda _2$ = 4.1858 +/− 0.0415Å) are computed. The selection of these two bandwidths is informed by the Bragg edges distributions of the two distinct powders. $\Delta \lambda _1$ encompasses the wavelength range where the Ni and Cu Bragg edges are both present. Conversely, $\Delta \lambda _2$ is limited to the wavelength range exclusively showcasing the Cu Bragg edge. Since we only applied a 3D CNN model for the beamline experiments, all *rQIs* are from our 3D CNN model, thus we omit the “CNN” notations in this section. Projection data from 40 neutron wavelengths bandwidths were binned and used to generate the individual reconstructions. The average $\overline{rQI_k^{\Delta \lambda }}$ corresponds to an average $\left( \frac{rQI_k^{\Delta \lambda _1} + rQI_k^{\Delta \lambda _2}}{2}\right)$, and is the reference value for the autonomous stopping criterion. Utilizing the average $\overline{rQI_k^{\Delta \lambda }}$ provides a representation of the overall quality across all bands. The inclusion of lower quality bands intentionally prevents a premature termination of the experiment by limiting the increase of $rQI_k^{\Delta \lambda _i}$. Simultaneously, higher quality bands help avoid redundant projections. This strategy provides a trade-off between image quality and the number of projections.

**Figure 7:**
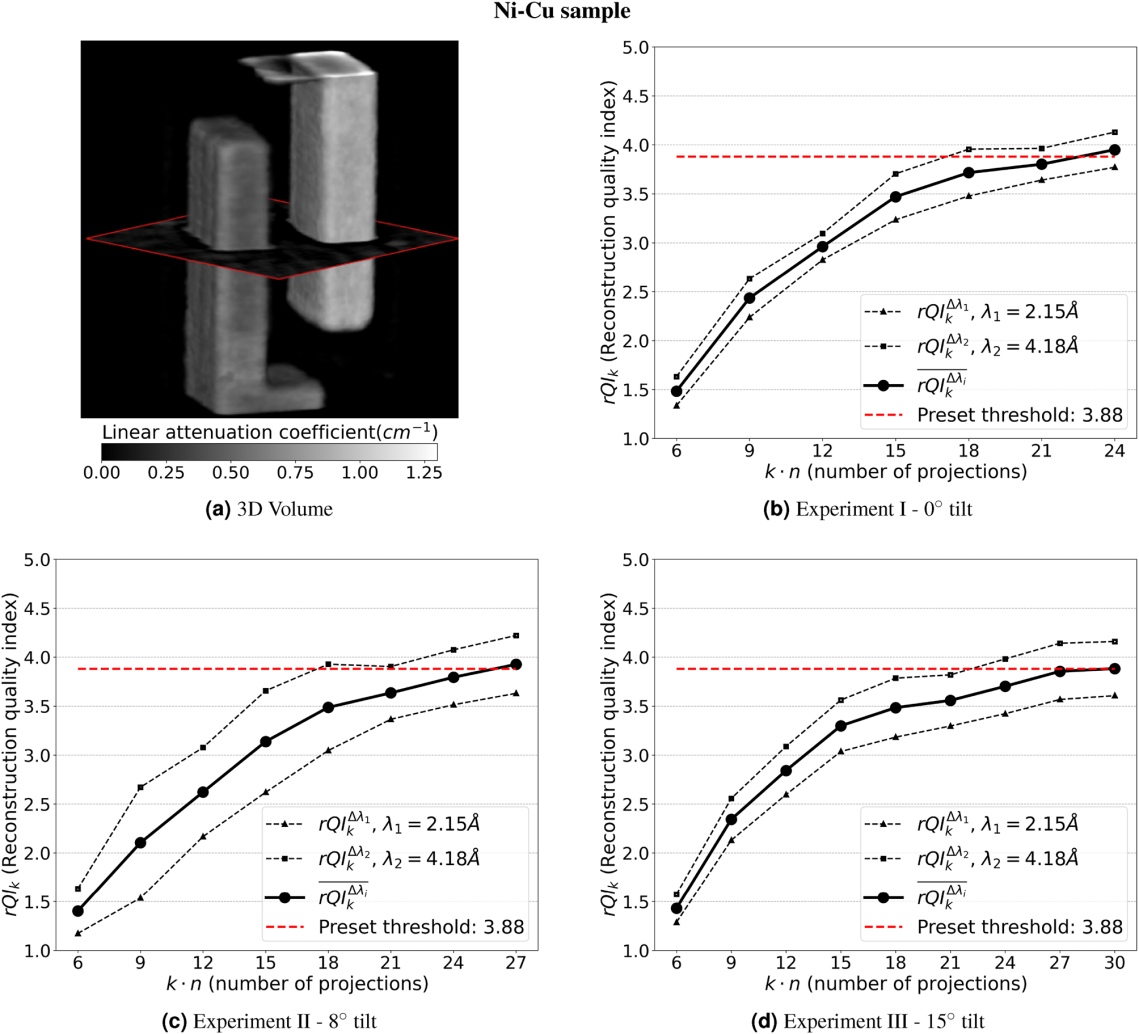
3D CNN rQIs values as a function of the number of projections for experiments I, II and III. In each figure, dashed lines are *rQIs* of reconstructions at 2.11 Å and 4.19 Å; the solid lines correspond to the averaged *rQI*. The red dashed horizontal lines are the rQI stopping value. (**a**) 3D reconstructed model of the Ni-Cu powder sample; (**b**) Exp. I: $0^{\circ }$ tilt; (**c**) Exp. II $8^{\circ }$ tilt; (**d**) Exp. III: $15^{\circ }$ tilt.

## Discussion

In materials science, as in other fields of research, the ability to scan a sample during in-situ experiments is essential in understanding the response of a novel material to stimuli. Although 2D radiographs provide some insights in the material’s behavior, they lack 3D information that is indispensable to understand the response of a material through its thickness and to also validate models which are often 3-dimensional. Hyperspectral neutron CT at spallation neutron sources provide unique wavelength-dependent contrast of crystalline and amorphous materials alike that is not common at reactor sources. However, spallation sources have lower integrated flux compared to their sister reactor facilities, making hyperspectral neutron CT a challenging task to accomplish in a few hours, let alone in minutes. Machine learning is an obvious technique for spallation sources due to the sheer amount of 3D hyperspectral neutron data that have less than desirable statistics. The focus of this manuscript was to develop an autonomous nCT workflow called HyperCT as the novel tool that not only decides projection angles to measure based on previous reconstruction knowledge^55^ but can also autonomously decide when a hyperspectral neutron CT scan has sufficient quality based on the selected region of interest of a sample (this manuscript). In the future, the automatic sample change or environmental parameter changes can be added into this autonomous workflow thereby expanding the utility of the proposed method to novel scientific studies.

As demonstrated in Fig. 5 and for the scoring system fed to the 3D CNN model during the “simulated” experiment (i.e., the values attributed to $\bar{q}_k$ based on the reconstructed data), there is no drastic improvement in the representative reconstructed slice after 25 projections for the NMC battery. In Appendix D, we assessed the reconstructed features by plotting the linear attenuation coefficient histogram of the object. As illustrated in the appendix, the distribution differences between the modeled and ground truth histograms decrease after 25 projections. This is the reason why the changes in the reconstructed data after $x_5$ (generated via 25 projections) become indistinguishable for the cathode. This observation serves as a potential basis to finish the hyperspectral neutron CT early. Further projections can be omitted as a pragmatic trade-off between measurement time and reconstruction quality. However, the scaffold slice continues to improve even though its *rQI* does not significantly change. (see Appendix D also for the histograms of the scaffold data with different number of projections.) This is due to the bias in training the 3D CNN and subjective assignment of quality score values. While a total number of 60 projections is sufficient to reconstruct a “solid” sample such as the NMC cathodes, it is insufficient to reconstruct a complex geometry such as the scaffold. This is also reflected by the fact that all quality indices for the cathodes are closer to each other (the *ACI* follows the $rQI^{GT}$ closer) than that of the scaffold. The $ACI_{k;k-1}$ plot shows a more “volatile” behavior than the $\bar{q}_k^{CNN}$ plot (see Fig. 5). This demonstrates why the individual quality indices ($\bar{q}_k^{CNN}$ and $ACI_{k;k-1}$) cannot represent well the changes in image quality for samples with different microstructures, thus the importance of our novel comprehensive quality index (*rQI*) to accurately reflect the image quality of a reconstructed data set while data is still being acquired, providing real time feedback to the user at the beamline. This is also further discussed in Appendix C. As demonstrated in Fig. 6, the reconstruction quality results are in good agreement with *rQI* values for the battery sample. However, improvement continues for the scaffold even though the *rQI* does not increase significantly, demonstrating that when samples have a more complex geometry (like the scaffold with its intricate and tortuous pathways), 60 projections are insufficient to be associated with a maximum score of $\bar{q}_k^{CNN}$ = 5. In another words, we need to include more training data for objects with complex structures.

Finally, Fig. 7 illustrates the performance of HyperCT for the real time hyperspectral neutron CT of a sample made of Ni and Cu powder. For the first time, a fully autonomous neutron CT experiment was performed at the SNS. The 0$^{\circ }$ tilt experiment I reaches the stopping criterion (*rQI* threshold), i.e. an acceptable image quality of the reconstructed data, in approximately 24 projections. This result is unparalleled in the field of hyperspectral neutron CT. Compared to the naïve scanning method (i.e. equally spaced projection angles and filtered-back projection algorithm for reconstruction) which may require several hundreds of projections to provide an acceptable reconstructed object, the proposed workflow drastically improves the efficiency of hyperspectral neutron CT. Moreover, experiment I requires less projections than the samples in the simulated experiments, i.e., the battery and scaffold. This may be due to the simpler geometry and higher homogeneity of the Ni-Cu, as compared to the previously mentioned samples. As the tilt increases, more projections are required due to the increase in neutron path length through the sample, thus reducing the overall transmission and thus increasing the measurement noise (see supplementary Appendix E for more details). Although SNS produces less intensity at longer wavelengths, such as $\Delta \lambda _2$ = 4.2 Å, than shorter wavelengths such as $\Delta \lambda _1$ = 2.2 Å, the rQI is never reached with the reconstruction performed at $\Delta \lambda _1$. This is due to the lack of contrast at $\Delta \lambda _1$, as compared to $\Delta \lambda _2$ = 4.2 Å for which samples exhibit significant contrast changes in transmission due to Bragg edges, thus yielding higher quality reconstructions and higher rQI values. (see Appendix E for more details).

## Conclusion

We have demonstrated the novel high-efficiency hyperspectral neutron CT workflow, HyperCT, which consists of several carefully designed features. We used svMBIR to complete a fast reconstruction with sparse projections. Specifically, svMBIR can complete a $512^3$ reconstruction within minutes using a multi-core computer consisting of 56 cores with hyper-threading. Such a high-speed reconstruction relative to the measurement times of a few hours per projection on commodity computing hardware is a critical piece of the autonomous loop. Compared to conventional FBP method, the reconstruction generated with svMBIR has much higher quality when using the same number of projections along with a intuitive way to set various parameters (see Appendix F).

The achieved HSnCT streaming system enables the design of measurement monitoring, quality evaluation and autonomous control for hyperspectral neutron CT experiments. This is a highly integrated system with a minimized learning curve for the end user. The users only need to choose an rQI threshold value and can launch the autonomous hyperspectral CT scan. The rQI not only evaluates the quality of a single real-time reconstruction using a 3D CNN model, but also measures the quality improvement between subsequent streaming reconstructions using the SSIM and NRMSE. This system also demonstrates the ability to increase the throughput at a neutron imaging beamline. Theoretically, a complete neutron CT that uses the ideal sampling rate^27^ requires a number of projections that is equal to 1.5$\times$ the number of detector pixels across the sample width on a radiograph. Thus, 200 projections are required to match the ideal sampling rate for the Ni-Cu At the SNS SNAP beamline, the neutron CT would require approximately 200-400 h, depending on the time spent for each hyperspectral projection. Using HyperCT, we were able to reduce to approximately 1/5 of the ideal sampling rate. This constitutes a “game changing” improvement of a factor> 5 in measurement time. In conclusion, HyperCT was successfully developed and implemented at the SNS, and can readily be demonstrated at other user facilities such as X-ray imaging beamlines at synchrotron sources.

### Supplementary Information

Supplementary Information.

## Data Availability

The experimental data that support the findings of this work are available from the corresponding authors upon reasonable request.
