# Supplementary material for: A machine learning decision criterion for reducing scan time for hyperspectral neutron computed tomography systems
Source: Sci Rep. 2024 Jul 2;14:15171. doi: 10.1038/s41598-024-63931-x (PMC11220078; doi:10.1038/s41598-024-63931-x)
Supplement: Supplementary file 1 — Supplementary Information. [file 41598_2024_63931_MOESM1_ESM.zip › SREP-24-00554-s10.pdf]

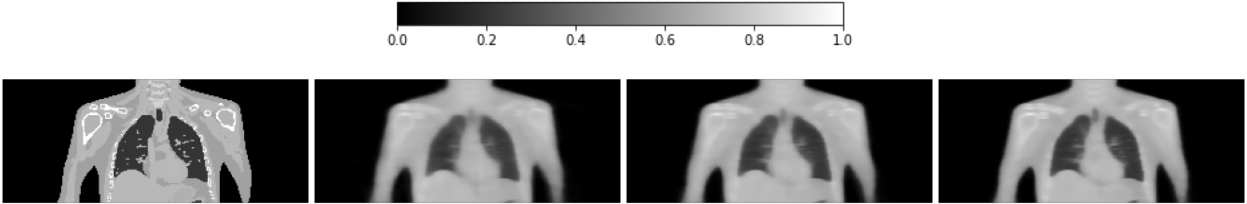

(a) Ground Truth

(b) Reconstruction from 15 projection

(c) Reconstruction from 20 projection

(d) Reconstruction from 30 projection
