# Supplementary material for: A machine learning decision criterion for reducing scan time for hyperspectral neutron computed tomography systems
Source: Sci Rep. 2024 Jul 2;14:15171. doi: 10.1038/s41598-024-63931-x (PMC11220078; doi:10.1038/s41598-024-63931-x)
Supplement: Supplementary file 1 — Supplementary Information. [file 41598_2024_63931_MOESM1_ESM.zip › SREP-24-00554-s12.pdf]

Appendix B

Cathode Sample Preparation and Cycling

The cathodes used in this manuscript were fabricated with Li ($Ni_{0.8}Mn_{0.1}Co_{0.1}$) O_2 (NMC811) from MSE Supplies[1] as the active material (AM) in an 80:10:10 wt% dry mixture of AM:Carbon Black (MSE Supplies):Polyvinylidene fluoride (PVDF) (Sigma Aldrich). Formation of the thick pellet cathodes was achieved by adding N-Methylpyrrolidone (NMP) to the dry mixture to create a thick slurry. The slurry was then cast into stainless steel washers of 10 mm internal diameter and 2 mm thickness and dried in ambient conditions for 48-72 hours. After ambient drying, the resulting pellet diameter was ~8 mm and thickness was ~1.3 mm. Once the initial drying was complete, the pellets were placed in a vacuum oven at 80°C for 12 hours and then transferred to an argon-filled atmosphere-controlled glovebox.

The thick pellets were assembled into half-cells in a Swagelok cell format with an electrolyte of 1 M $LiPF_6$ in ethylene carbonate-diethyl carbonate. Lithium foil was applied as the counter electrode. The cells were cycled from 3.0 to 3.9V vs. Li/Li+ at a current of 20μA using a BioLogic Science Instruments VSP potentiostat/galvanostat and held at 3.9V for 24 hours between each cycle. Following cycling the test cells were disassembled in an argon-filled glovebox and the cathodes were dried for 72 hours. After drying the cycled cathodes and one pristine cathode were stacked into a Teflon sleeve with Teflon washers positioned between each sample. The sample holder was sealed with Kapton tape for subsequent neutron imaging studies.

Scaffold sample preparation

The scaffold used in this manuscript was manufactured for use as a bone filler for the regeneration of bone defects and voids. The scaffold is composed of polyurethane (PU), nanohydroxyapatite (nHA), and decellularized bone particles (DBP). The polymer PU was mixed with nHA (PU-nHA ratio: 80/20 w/w) and then integrated into a 3D porous structure by including DBP to provide mechanical stability. The physical and mechanical properties were confirmed using X-ray diffraction, X-ray photon electron spectroscopy and thermogravimetric analysis. The surface properties of the scaffold was evaluated using atomic force microscopy, nano-indentation and contact angle measurement[2-4]. The scaffold was cut into 1×1×1mm section and imaged using neutron computed tomography to determine structural properties.

References

1 *MSE Supplies*, <https://www.msesupplies.com>

2 Alghazali, K. M. *et al.* Bone-tissue engineering: complex tunable structural and biological responses to injury, drug delivery, and cell-based therapies. *Drug metabolism reviews* **47**, 431-454 (2015).

3 Jackson, B. K. *et al.* Polyurethane/nano-hydroxyapatite composite films as osteogenic platforms. *Journal of Biomaterials science, Polymer edition* **29**, 1426-1443 (2018).

4 Bow, A. *et al.* Evaluation of a polyurethane platform for delivery of nanohydroxyapatite and decellularized bone particles in a porous three-dimensional scaffold. *ACS Applied Bio Materials* **2**, 1815-1829 (2019).
