# Supplementary material for: A machine learning decision criterion for reducing scan time for hyperspectral neutron computed tomography systems
Source: Sci Rep. 2024 Jul 2;14:15171. doi: 10.1038/s41598-024-63931-x (PMC11220078; doi:10.1038/s41598-024-63931-x)
Supplement: Supplementary file 1 — Supplementary Information. [file 41598_2024_63931_MOESM1_ESM.zip › SREP-24-00554-s13.pdf]

Appendix C

Performance comparison between BRISQUE and 3DCNN in Quality Score Estimation

To evaluate the performance of our proposed 3D CNN quality score indicator, rQI, we use the Blind/Referenceless Image Spatial QUality Evaluator (BRISQUE)[1]. BRISQUE is trained with the same neutron data to provide its performance baseline. BRISQUE is a traditional method for no-reference image quality assessment. In this research, the same cuboids used for the CNN training are utilized for the BRISQUE training, with the cuboids converted into slices since BRISQUE is a 2D evaluator. All slices of the cuboid have the same label as the 3D cuboid. BRISQUE is applied to each slice of the 3D cuboid and the average estimated score is the final cuboid quality score.

The corresponding confusion matrices are shown in Table B1. A confusion matrix represents the performance of a machine learning algorithm by comparing the predicted class to the actual class in a matrix form. The first row corresponds to the predicted labels, and the first column shows the ground truth labels. The matrix displays how many samples are correctly and incorrectly predicted per class, and thus illustrates the classes that are being confused as other classes by the model. The ith row represents the percentage of the samples of the ith class classified into each class. Therefore, the diagonal cells are the percentage of samples that are classified correctly. Other cells are percentages of misclassified samples. The table shows results for both the BRISQUE and 3D CNN models.

Based on the confusion matrix of both models, more samples from classes 2 and 4 are misclassified into classes 3 and 5. The BRISQUE model has a higher overall misclassification rate into the neighboring classes as compared to our CNN model. The 3D CNN model only misclassifies a small number of samples into the neighboring classes. For example, 1.51% of the samples belonging to class 1 are misclassified into class 2. The BRISQUE model also misclassifies 0.02% of class 1 into class 3 samples. Moreover, the 3D CNN model classifies more samples correctly than the BRISQUE model. The 3D CNN model performs better than the BRISQUE model.

There are 9.47% class 5 samples that are misclassified into class 4, which is relatively high compared to other CNN models. This may illustrate that the reconstructions from 45 and 60 projections are similar, which means redundant information was acquired after the first 45 projections. We emphasize that the results are only demonstrated for sub-sampled 3D volumes of the object.

Table C1 Confusion matrices of the BRISQUE (top) and 3DCNN (bottom) models: The models are trained using 4300 cuboids of each score. These cuboids are extracted from both the battery cathode and scaffold samples. Predicted quality scores (columns) are compared to ground truth scores (rows). More than 86.3% of the scores are predicted correctly for the BRISQUE model, whereas our 3DCNN model reaches a higher prediction > 90.5%. Thus, the results illustrate that the 3DCNN model is more accurate than the BRISQUE model.

			**Predicted**				
		Projection number	*5*	*10*	*20*	*45*	*60*
		**Quality Scores**	***1***	***2***	***3***	***4***	***5***
**Ground Truth**	BRISQUE	**1**	**98.79%**	1.19%	0.02%	0	0
		**2**	11.72%	**87.89%**	0.40%	0	0
		**3**	0.16%	13.37%	**86.30%**	0.16%	0
		**4**	0	0.09%	4.89%	**94.90%**	0.12%
		**5**	0	0	0.19%	12.95%	**86.87%**
	3D CNN	**1**	**98.49%**	1.51%	0	0	0
		**2**	1.63%	**98.16%**	0.21%	0.0%	0
		**3**	0	2.40%	**97.48%**	0.12%	0
		**4**	0	0	0.07%	**96.92%**	3.01%
		**5**	0	0	0	9.47%	**90.53%**

We also demonstrate the performance of the two models using full-size reconstructions, where the quality score is the mean value of all non-overlapping cuboids. Figure C1 shows the computed quality scores as a function of *kn* (the number of projections*)* for both models, where $\bar{q}_k^{CNN}$ and $\bar{q}_k^{BRISQUE}$ are the averaged 3D CNN and BRISQUE models output quality reconstruction score using kn projections, respectively.

We calculated the RMSE between estimated scores $\bar{q}_k$ and subjectively assigned scores (considered the ground truth values) q_k^{GT} to illustrate the overall difference. The RMSE is given by:

$$RMSE = \sqrt{\frac{1}{K}\sum_{k=1}^{K}(\bar{q}_k - q_k^{GT})^2} \qquad \text{(C-a)}$$

where K is the number of reconstructions included in the plot.

Figure C1 displays the averaged and ground truth quality scores as a function of the number of projections for the battery and scaffold samples. The RMSE for each model is also shown on the figure. As displayed in the figure, both models behave similarly and are close to the ground truth quality score for the battery sample. However, the accuracy and robustness of our 3D CNN model at estimating the quality score of a full-size CT reconstruction surpasses those of the BRISQUE model. The 3D CNN quality scores follow the ground truth curve closer than the BRISQUE model scores below 30 projections. The simulated RMSEs of the cathode are generally lower than the RMSEs of the polymer, which may be due to the prominent edges of the battery, which are easier to extract and to learn from. The scaffold sample is much more complex and thus more difficult to capture during the model training process. Additionally, the cathode sample occupy more voxels in the reconstruction compared to the scaffold sample. Hence, the SNR is lower for the scaffold than the cathode which introduces more noise when computing the quality scores.

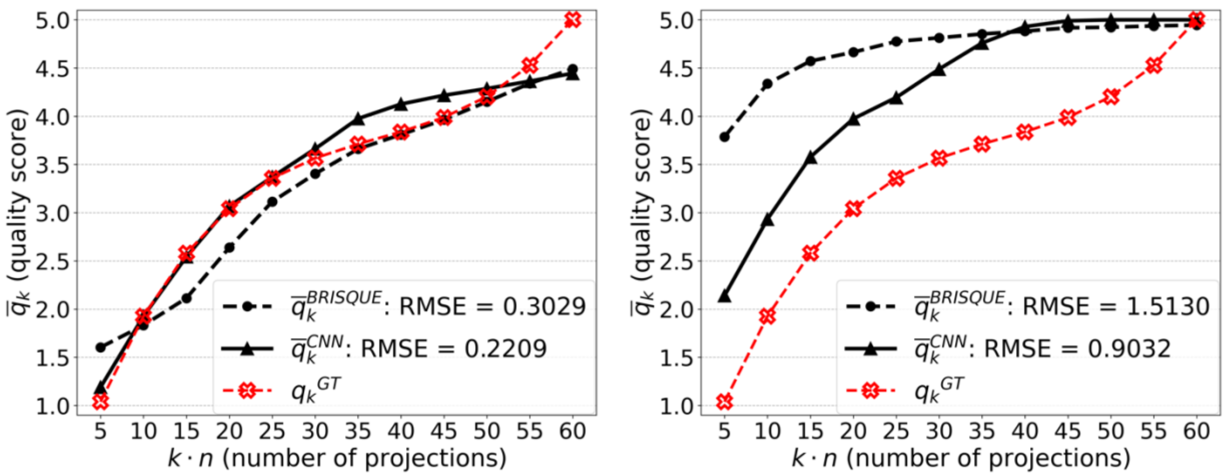

(**a**) NMC cathode (**b**) Polymer scaffold

Figure C1. Estimated quality scores of the simulated streaming CT as a function of the number of projections, using the BRISQUE and 3DCNN models, respectively, for (a) the NMC cathode, and (b) the polymer scaffold. The red crosses represent the ground truth quality score. These results show that for the cathode, both models are similar to the ground truth, yielding low RMSE values. However, for the polymer sample, the BRISQUE model quality score overestimates further from the ground truth than our model. In this case, both models reach a plateau at 35 projections. Similarly, the RMSE is higher for the BRISQUE model than our CNN model, indicating that our model offers a quality score that better represents the ground truth.

Figure C2 displays the quality indices, $\mathrm{rQI}^{\mathrm{GT}}$, $rQI_k^{BRISQUE}$, $\bar{q}_k^{BRISQUE}$, and $ACI_{k;k-1}$, as a function of *kn* for the NMC cathode and the polymer scaffold, respectively. We also plotted the indices ($\bar{q}_k^{BRISQUE}$ and $rQI_k^{BRISQUE}$) from the BRISQUE model for the corresponding reconstruction generated by *kn* projections in Figure C2, where the x-axis is the number of projections, and the y-axis corresponds to the quality indices. Additionally, the $ACI_{k;k-1}$ is also plotted as a function of the number of projections. As expected, the $rQI_k^{BRISQUE}$ increases rapidly with the number of projections up to a value of 3.5, at which point the index values do not increase significantly. Due to the larger disparity in the $\bar{q}_k^{BRISQUE}$, the $rQI_k^{BRISQUE}$ has a larger RMSE compared to the rQI_k^{CNN} RMSE shown in Figure 5 from the manuscript (values were 0.1381 and 0.5290 for the cathode and scaffold RMSEs, respectively).

Similar to the 3D CNN model, $rQI_k^{BRISQUE}$ is in better agreement with the $\mathrm{rQI}^{\mathrm{GT}}$ for the NMC cathode than for the scaffold due to the lack of prominent edges in the polymer sample. The difference in performance with the two datasets highlights that the assessment is not a universal method. Particularly in the polymer scaffold, $rQI_k^{BRISQUE}$ remains further away from the ground truth when using fewer projections for the reconstruction. A higher RMSE indicates that the BRISQUE model performs poorly when the reconstruction quality is suboptimal, possibly due to the lower ratio of sample pixels to background pixels introducing noise into the assessment.

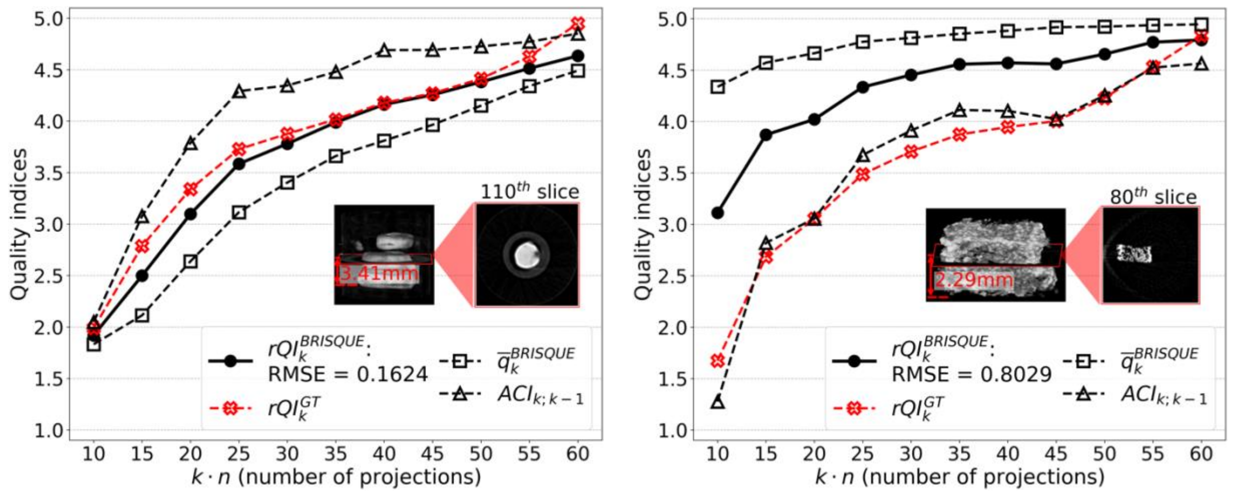

(a) NMC cathode (b) Polymer scaffold

Figure C2. Quality indices ($rQI_{k;k-1}$, q_k, and $ACI_{k;\,k-1}$) as a function of the number of projections used for the reconstruction during the simulated experiment. Solid lines represent the rQIs, and the dashed lines are the quality scores, q_k, and ACI. The red crosses correspond to the (user-defined) ground truth rQI^{GT}s. Indices are computed using the BRISQUE model for the (a) NMC battery electrode and (b) polymer scaffold. An example slice from the corresponding reconstruction is also displayed in the plot.

References

1 Mittal, A., Moorthy, A. K. & Bovik, A. C. in *2011 Conference Record of the Forty Fifth Asilomar Conference on Signals, Systems and Computers (ASILOMAR).* 723-727.
