# Supplementary material for: A machine learning decision criterion for reducing scan time for hyperspectral neutron computed tomography systems
Source: Sci Rep. 2024 Jul 2;14:15171. doi: 10.1038/s41598-024-63931-x (PMC11220078; doi:10.1038/s41598-024-63931-x)
Supplement: Supplementary file 1 — Supplementary Information. [file 41598_2024_63931_MOESM1_ESM.zip › SREP-24-00554-s14.pdf]

		Predicted					
		Projection number	*5*	*10*	*20*	*45*	*60*
		**Quality Scores**	***1***	***2***	***3***	***4***	***5***
**Ground Truth**	BRISQUE	**1**	**98.79%**	1.19%	0.02%	0	0
		**2**	11.72%	**87.89%**	0.40%	0	0
		**3**	0.16%	13.37%	**86.30%**	0.16%	0
		**4**	0	0.09%	4.89%	**94.90%**	0.12%
		**5**	0	0	0.19%	12.95%	**86.87%**
	3D CNN	**1**	**98.49%**	1.51%	0	0	0
		**2**	1.63%	**98.16%**	0.21%	0.0%	0
		**3**	0	2.40%	**97.48%**	0.12%	0
		**4**	0	0	0.07%	**96.92%**	3.01%
		**5**	0	0	0	9.47%	**90.53%**
