# Supplementary material for: A machine learning decision criterion for reducing scan time for hyperspectral neutron computed tomography systems
Source: Sci Rep. 2024 Jul 2;14:15171. doi: 10.1038/s41598-024-63931-x (PMC11220078; doi:10.1038/s41598-024-63931-x)
Supplement: Supplementary file 1 — Supplementary Information. [file 41598_2024_63931_MOESM1_ESM.zip › SREP-24-00554-s19.pdf]

Appendix D

Statistical Comparison of Histograms Obtained from the Ground Truth and "Simulated Experiment" Data

We employed a straightforward bi-class K-means classifier to effectively delineate the object region from the background during reconstruction. Subsequently, we identified the contour of the object as a mask and applied a masking operation to isolate the voxels within reconstructed objects. Since the reconstructed voxel values are proportional to the linear attenuation coefficient, the histogram of voxel values within the object effectively captures the object's response to neutrons.

To statistically compare the reconstructed and ground truth histograms, we calculated the Spearman correlation coefficients[1] and Wasserstein distance[2] between both histograms using white beam data (no wavelength discrimination). The Spearman correlation is particularly advantageous when the relationship between variables is not linear or when the data contains outliers. It is a rank-based measure, and it focuses on the order or ranking of the data rather than their specific numerical values. The formula for the Spearman correlation coefficient is as follows:

$$\rho = 1 - \frac{6\sum d_i^2}{n(n^2-1)} \qquad \text{(D a)}$$

where d_i is the difference between the ranks of corresponding values, and n is the number of data points. $\rho = 1$ corresponds to a perfect monotonic increasing relationship between the two histograms. Otherwise $\rho = 0$ indicates no monotonic relationship.

The Wasserstein distance, also known as the Earth Mover's Distance (EMD) or Kantorovich-Rubinstein distance, is a metric used to quantify the distance between two probability distributions ($P_r(x)$ and $P_\theta(y)$) over a metric space. It provides a measure of the minimum "cost" required to transform one distribution into another, where the cost is determined by the product of the distance between points ($\|x-y\|$) in the metric space and the amount of mass transported between those points ($\sum_{x,y}\gamma(x,y)$):

$$W(P_r, P_\theta) = \inf_{\gamma \sim \Pi(P_r, P_\theta)} \sum_{x,y} \gamma(x,y)\|x-y\| \qquad \text{(D b)}$$

Figure D1 represents the comparison between the histogram distributions of the reconstructed (for different numbers of projections) and the ground truth data for the NMC cathode. The ground truth histogram remains unchanged since it always uses all 60 projections. The histogram of the simulated experiment becomes increasingly similar to the ground truth as the number of projections increases. After 25 projections, both histograms become similar. This observation confirms that the reconstruction quality does not improve significantly after 25 projections for the NMC cathode sample.

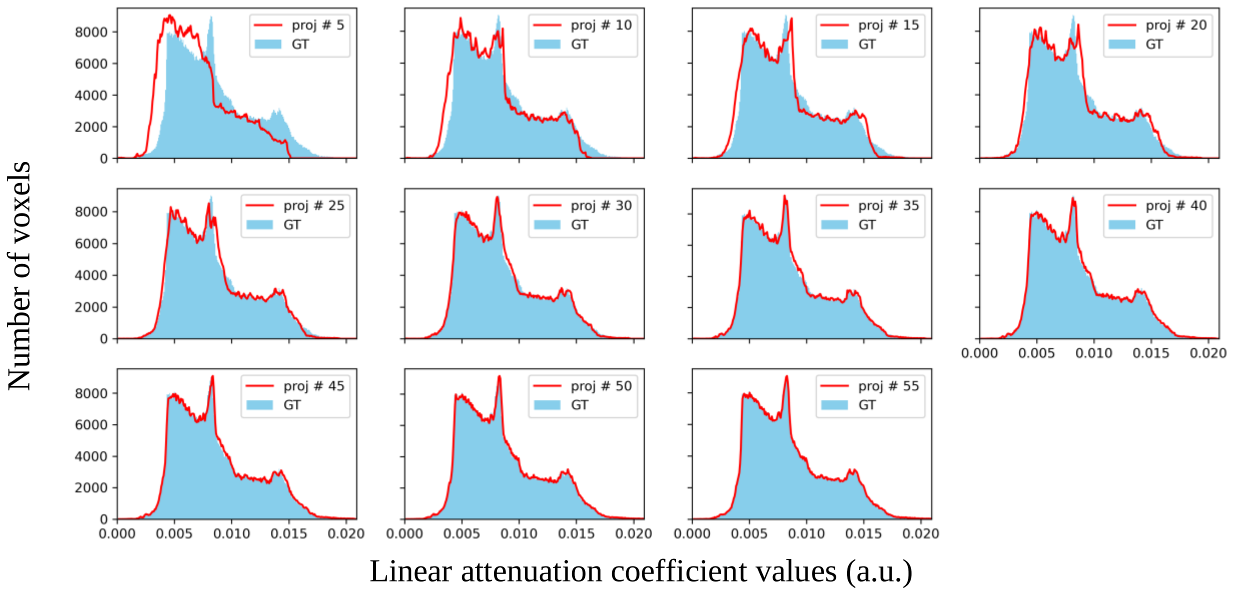

Figure D1. Comparative histograms, i.e., number of voxels vs. linear attenuation coefficient values, of the simulated experiment and the ground truth for the NMC cathode. The red line is the histogram of the reconstruction corresponding to the number of projections indicated in the top right corner of each plot. The blue shape is the unchanged ground truth histogram based on the reconstruction that used all 60 projections and denoted as GT in the legend. The histograms become similar after 25 projections, indicating that there is no significant improvement in the reconstructed data after 25 projections.

Figure D2 illustrates the assessment of the similarities between the simulated experiment and ground truth histograms. Figures D2(a) and D2(b) display the Spearman correlation and the Wasserstein distance values, respectively, as a function of the number of projections used for the reconstruction of the NMC cathode. As illustrated in Figure D2(a), the Spearman correlation converges to 1 with the increasing number of projections. The Spearman correlation does not increase significantly after 30 projections, at which point it is close to 1. Similarly, the Wasserstein distance decreases drastically until 30 projections. This analysis suggests that the quality of reconstruction does not change after 30 projections. Considering the compromise between measurement time and reconstruction quality, we conclude that for the NMC cathode, 25-30 projections are sufficient for the reconstruction.

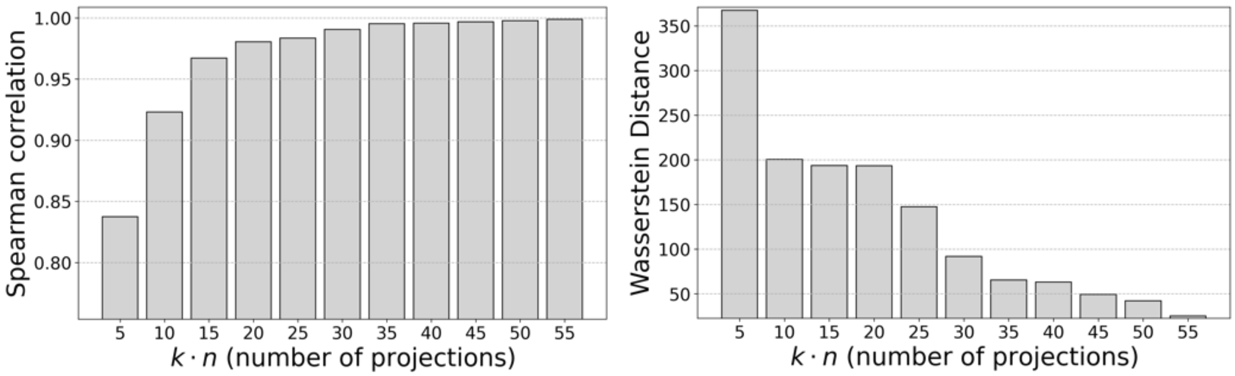

(a) (b)

Figure D2 Assessment of the similarities between the histograms for the simulated experiment and the ground truth for the NMC cathode: (a) the Spearman correlation coefficient and (b) the Wasserstein distance, respectively, as a function of the number of projections. The Spearman correlation does not significantly increase after 25 projections and is close to 1. Similarly, after 25 projections, the Wasserstein distance does not decrease considerably.

The same analysis was performed with the scaffold data, as illustrated by the linear attenuation coefficient histograms of the streaming reconstructions in Figure D3, and the corresponding similarity evaluations in Figure D4.

We utilized the reconstruction generated with 60 projections as the ground truth histogram. In Figure D3, the histograms of the simulated experiments and the ground truth are plotted as a function of the number of projections used for the reconstruction. The histograms remain dissimilar until the number of projections has reached a value of 55, which is well above the 25 projections observed with the NMC cathode. This illustrates how complex structures (like a scaffold) require more projections for our novel method HyperCT to work.

Figures D4(a) and D4(b) display the Spearman correlation and the Wasserstein distance values, respectively, as a function of the number of projections used for the reconstruction of the scaffold. Although the Spearman correlation does not significantly increase after 40 projections and is close to 1, the Wasserstein distance continues to decrease after 40 projections. This indicates that for complex geometries, the Wasserstein distance may be a better assessment of the histogram similarities than the Spearman correlation. In light of this supplementary information, we claim that the optimal projection number corresponding to the highest subjective score must be tailored on a per-object basis. Specifically, for objects with a more intricate structure, a higher number of projections may be warranted.

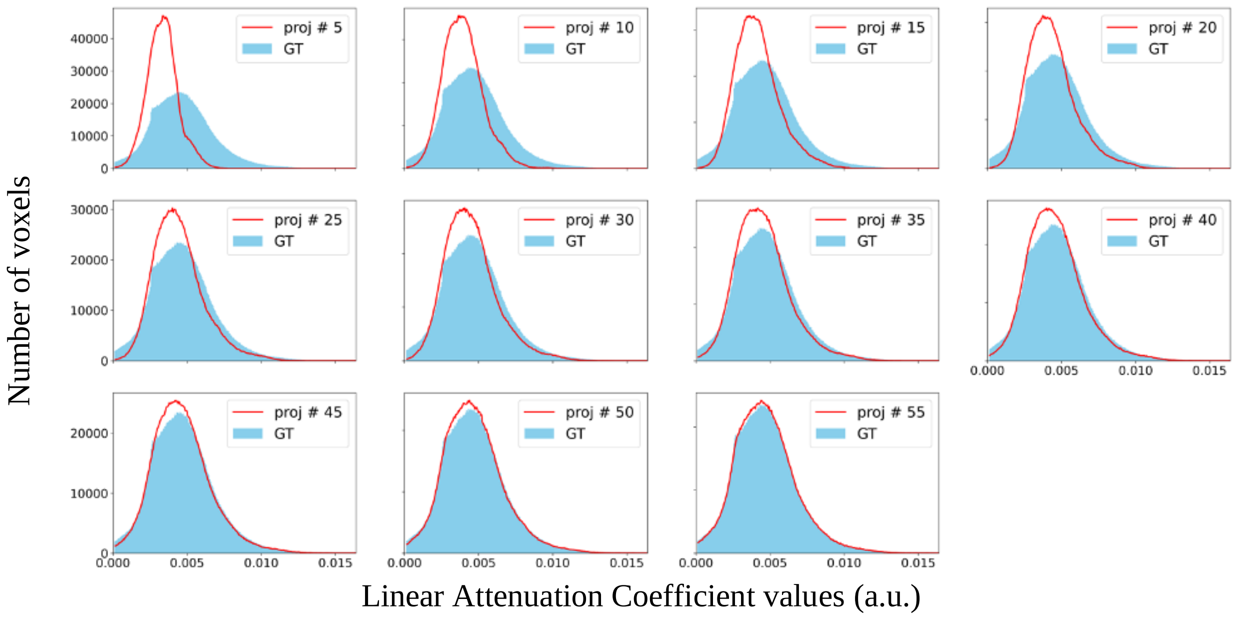

Figure D3. Comparative histograms, i.e., number of voxels vs. linear attenuation coefficient values, of the simulated experiment and the ground truth for the scaffold. The red line is the histogram of the reconstruction corresponding to the number of projections indicated in the top right corner of each plot. The blue shape is the unchanged ground truth histogram based on the reconstruction that used all 60 projections and denoted as GT in legend. The histograms become similar after 55 projections, indicating that more projections are needed for our method to work when the sample has a more complex geometry.

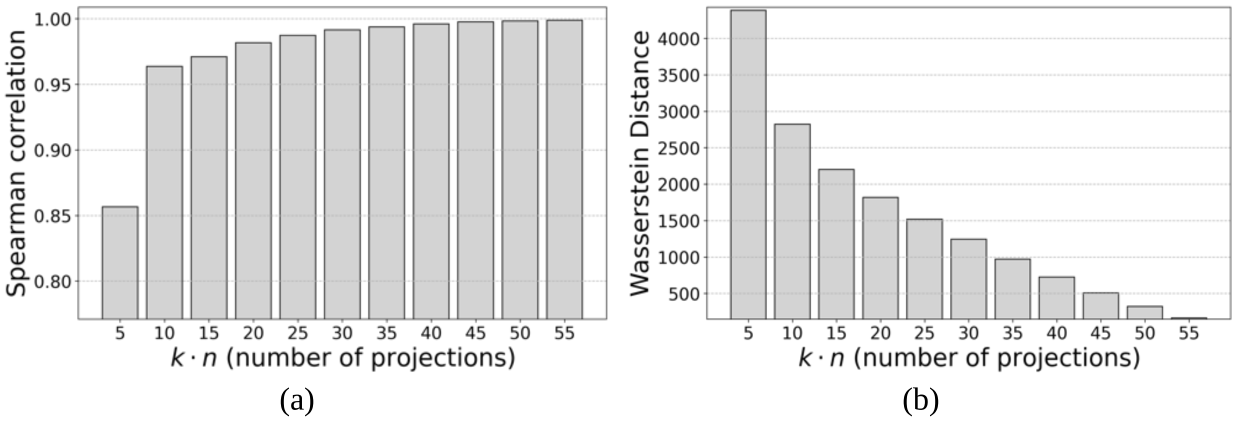

Figure D4 Assessment of the similarities between the histograms for the simulated experiment and the ground truth for the scaffold sample: (a) the Spearman correlation coefficient and (b) the Wasserstein distance, respectively, as a function of the number of projections. Although the Spearman correlation does not significantly increase after 40 projections and is close to 1, the Wasserstein distance continues to decrease after 40 projections. This indicates hat for complex geometries, the Wasserstein distance may be a better assessment of the histogram similarities than the Spearman correlation.

Reference

1 Spearman, C. The proof and measurement of association between two things. *International Journal of Epidemiology* **39**, 1137-1150 (2010). https://doi.org:10.1093/ije/dyq191

2 Kantorovich, L. V. On the Translocation of Masses. *Journal of Mathematical Sciences* **133**, 1381-1382 (2006). https://doi.org:10.1007/s10958-006-0049-2
