# Supplementary material for: A machine learning decision criterion for reducing scan time for hyperspectral neutron computed tomography systems
Source: Sci Rep. 2024 Jul 2;14:15171. doi: 10.1038/s41598-024-63931-x (PMC11220078; doi:10.1038/s41598-024-63931-x)
Supplement: Supplementary file 1 — Supplementary Information. [file 41598_2024_63931_MOESM1_ESM.zip › SREP-24-00554-s24.pdf]

Appendix E

Elements Influencing the Quality of the Hyperspectral Neutron Computed Tomography

We computed the signal-to-noise ratio (SNR) using the Ni-Cu sample data as the basic quality matrices of the normalized projections. The SNR is calculated using the summed pixel values of the selected sample area divided by the background. Figure E1 displays the normalized transmission projections of the Ni-Cu sample for the three vertical tilt positions and the selected pixels used to calculate the SNR displayed in Figure E2. The figure shows that the SNRs are on average higher at 2.15 Å and depend on the projection angle (i.e. sensitivity to the change of neutron path through the sample as it rotates). For all projections, the sample without tilt has better SNR and the ones with tilt, further illustrating the negative effect of the increase of the sample thickness through the beam.

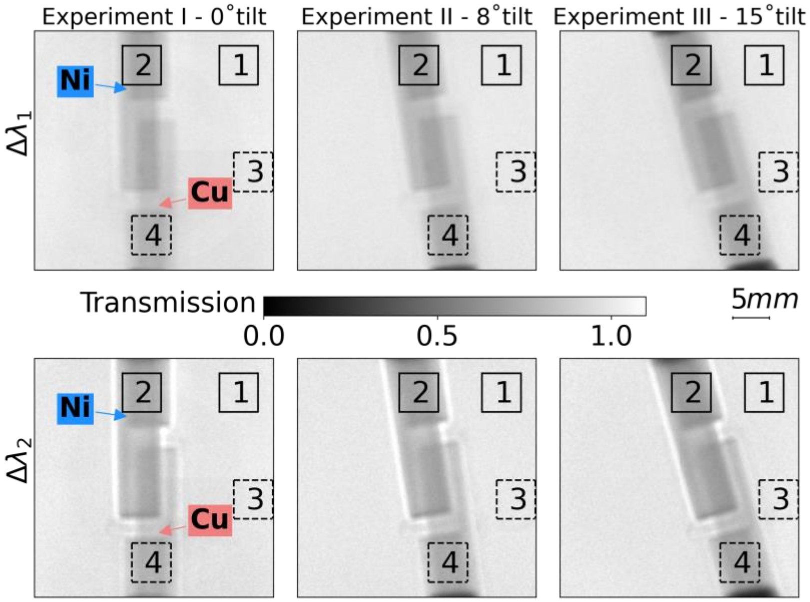

Figure E1. Normalized radiographs of the Ni-Cu sample showing the selected regions of interest (ROIs) for each experiment (the projection angle is 91.184˚): ROIs 1 and 3 correspond to the background; ROIs 2 and 4 correspond to the Ni and Cu samples, respectively.

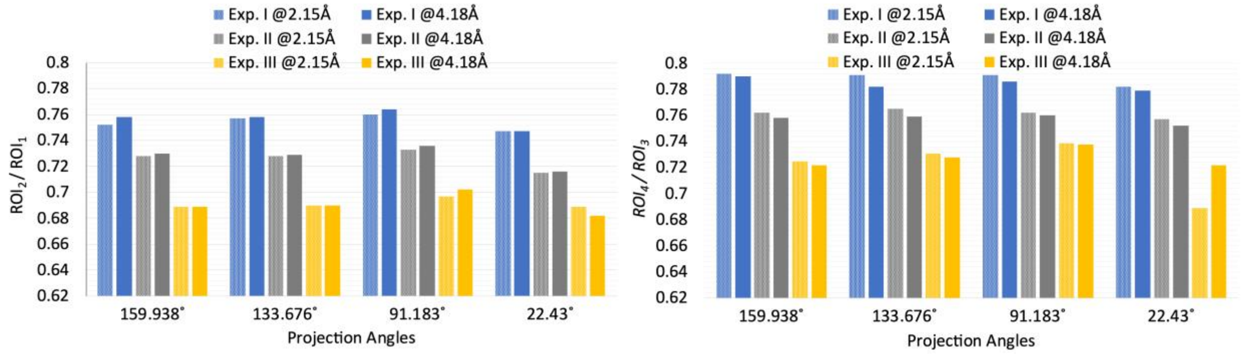

(a) (b)

Figure E2. SNR values at 2.15Å and 4.18Å for the projection angles shown in Figure E1: (a)ROI_2/ROI_1, and (b) ROI_4/ROI_3.The figure shows that the SNRs are on average higher at 2.15 Å, and depend on the projection angle (i.e. sensitivity to the change of neutron path through the sample as it rotates). For all projections, the sample without tilt has better SNR than the ones with tilt, further illustrating the negative effect of the increase of the sample thickness through the beam.

Figure E3 displays representative reconstructed slices of the Ni-Cu sample at 2.15Å and 4.18 Å (values chosen based on Bragg edges, see Figure E4), respectively, along with their rQI values. Although the reconstructions were performed using the same number of projections, the reconstruction has better quality at 4.18Å than at 2.15Å based on the rQI values and the overall image quality of the slices.

Figure E4 illustrates the transmission values of Cu and Ni as a function of neutron wavelength. The Cu powder has transmission Bragg edges close to 4 .18Å, which increases the contrast in the reconstruction, hence improving the reconstruction quality. The image quality is similar at both wavelengths, however the corresponding rQI values are lower for the 2.5 Å data than the 4.18 Å, indicating that the overall count rate being higher at shorter wavelengths plays a significant role in the reconstruction quality. The image quality of the slices improves with the increase of rQI values.

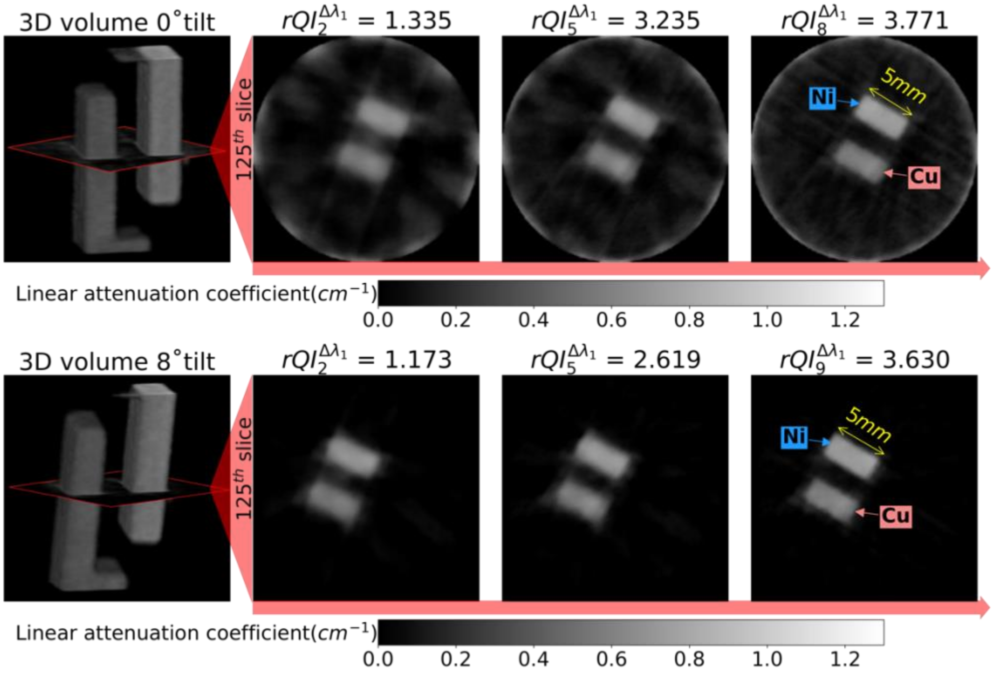

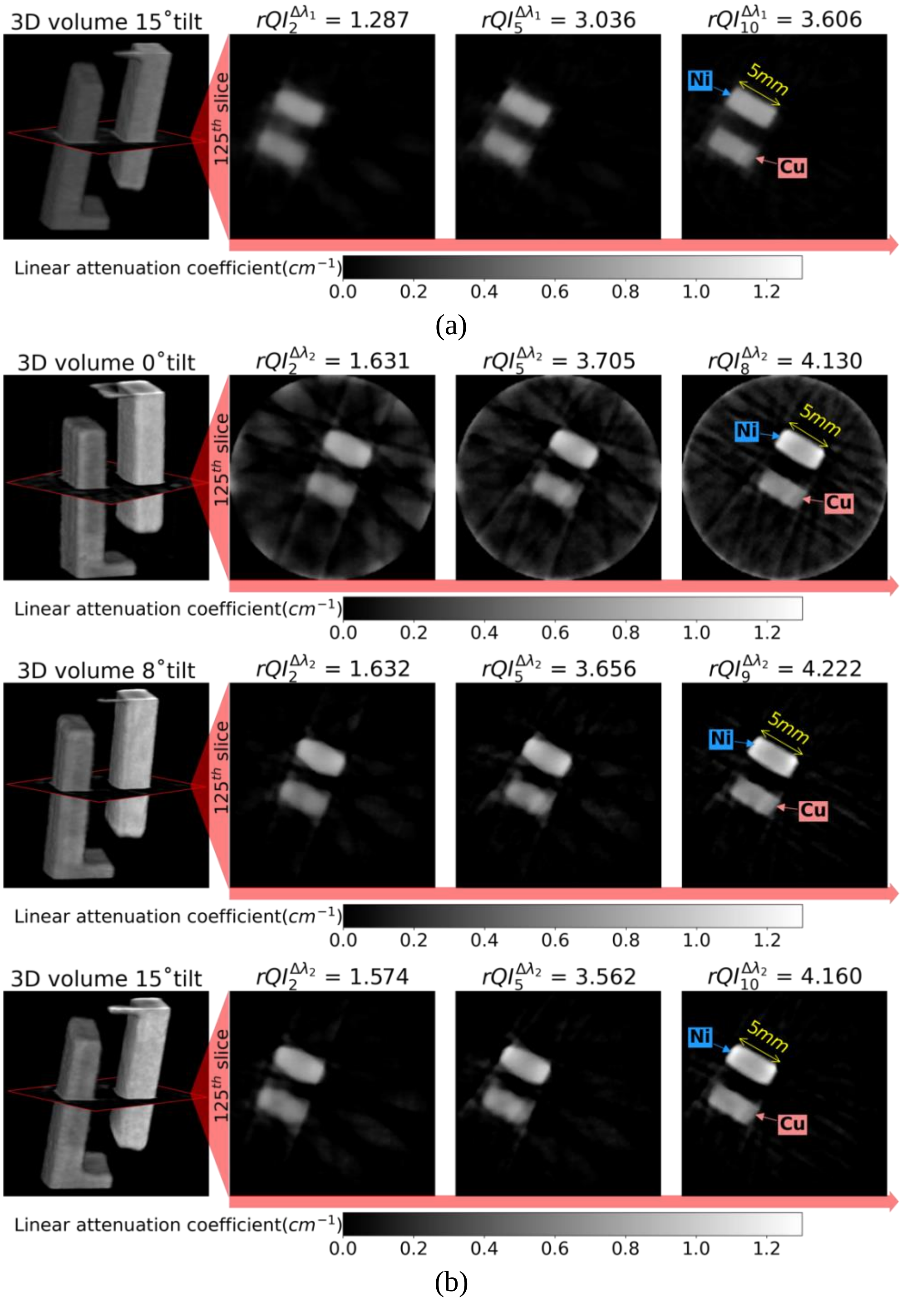

Figure E3. Representative reconstructed slices of the Ni-Cu sample with their respective rQI values: (top) Exp. I (no-tilt), (center) Exp. II (8˚-tilt), and (bottom) Exp. III (15˚-tilt). The left and right columns correspond to reconstructions performed at (a) 2.15 Å and (b) 4.18 Å, respectively. The image quality is similar at both wavelengths, however the corresponding rQI values are lower for the 2.5 Å data than the 4.18 Å ones, indicating that the overall count rate being higher at shorter wavelengths does not play a significant role in the reconstruction quality as compared to a higher contrast at longer wavelengths due to the proximity of a Bragg edge. The image quality of the slices improves with the increase of rQI values.

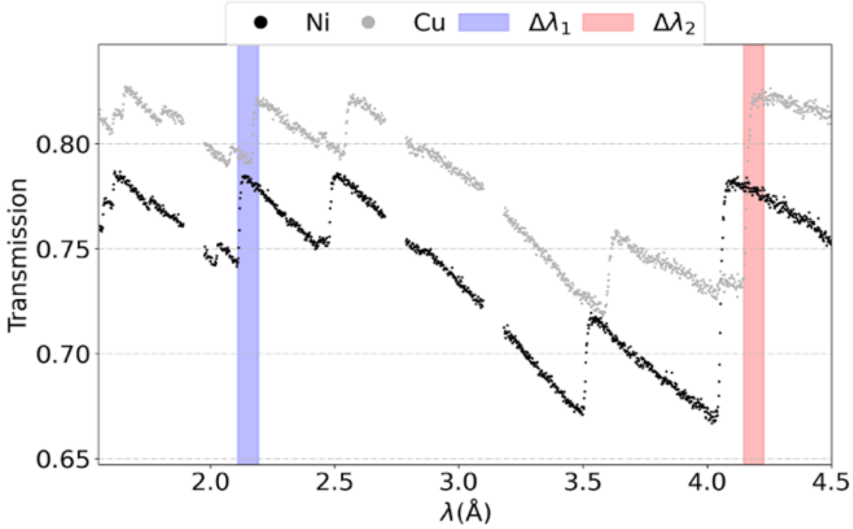

Figure E4. Transmission as a function of neutron wavelength for the Ni and Cu powders showing the so-called Bragg edge. The figure also shows the two narrow wavelength bins used of the reconstructions displayed in Figure E3. While the shorter wavelength bin contains Bragg edges for both powders, the longer wavelength bin is chosen to match the Cu Bragg edge.
