# Supplementary material for: A machine learning decision criterion for reducing scan time for hyperspectral neutron computed tomography systems
Source: Sci Rep. 2024 Jul 2;14:15171. doi: 10.1038/s41598-024-63931-x (PMC11220078; doi:10.1038/s41598-024-63931-x)
Supplement: Supplementary file 1 — Supplementary Information. [file 41598_2024_63931_MOESM1_ESM.zip › SREP-24-00554-s25.pdf]

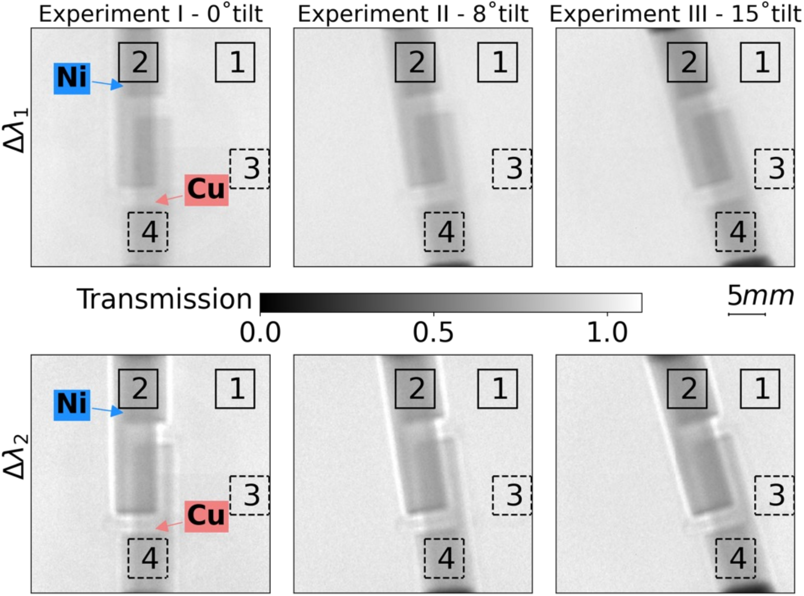

Experiment I - 0°tilt
Experiment II - 8°tilt
Experiment III - 15°tilt
Δλ1
Δλ2
Ni
Cu
1
2
3
4
Transmission
0.0
0.5
1.0
5mm
