# Supplementary material for: A machine learning decision criterion for reducing scan time for hyperspectral neutron computed tomography systems
Source: Sci Rep. 2024 Jul 2;14:15171. doi: 10.1038/s41598-024-63931-x (PMC11220078; doi:10.1038/s41598-024-63931-x)
Supplement: Supplementary file 1 — Supplementary Information. [file 41598_2024_63931_MOESM1_ESM.zip › SREP-24-00554-s30.pdf]

Appendix F

Comparison between svMBIR and FBP reconstruction methods

One of the key strengths of our approach is the use of a model-based iterative reconstruction algorithm at each stage. MBIR methods are known to be able to produce high quality reconstruction from sparse-view, irregular and low SNR data sets compared to the traditional FBP algorithm. Next, we provide some details about MBIR, our specific software implementation of MBIR and how it compares to FBP.

In MBIR, the 3D reconstruction is cast as solving an optimization problem of the form,

$$c(x) \leftarrow \underset{x}{\operatorname{argmin}}\{\frac{1}{2\sigma_y^2}\|y - Ax\|_W^2 + \sum_{i,j\in\chi} w_{i,j}\rho(\frac{x_i - x_j}{\sigma_x})\} \quad \text{F-a}$$

where A is the forward projection matrix, y is the appropriately normalized projection data, W is a diagonal inverse noise co-variance matrix, $w_{i,j}$ is a kernel that assigns a lower weight to neighboring voxels that are far apart, ρ is a penalty function used to enforce spatial correlations and σ_x and σ_y are parameters that can be adjusted to control the noise or resolution in the final reconstruction.

An often ignored topic in MBIR is the selection of the regularization parameters σ_y, and σ_x in order to obtain a satisfactory reconstruction. In practice these values are chosen empirically often to obtain a reasonable visual image quality i.e. balancing noise and resolution in the reconstruction. While this approach is used, choosing these parameters is often un-intuitive to end users of various CT systems. In order to alleviate this issue, we use the svMBIR library[1]. The svMBIR library, is one of the first efforts where the package provides automatic reasonable initial estimates for these parameter values using the following relations, based on two user inputs, the typical SNR of the raw data (σ_n) and the desired sharpness in the reconstruction (S). Specifically,

$$\hat{\sigma}_y \leftarrow \sigma_n \sigma_S \quad \text{F-b}$$

Where

$$\sigma_S = \sqrt{\frac{1}{\widetilde{M}}\sum_{i=1}^{\widetilde{M}} W_{ii} b_i y_i^2} \quad \text{F-c}$$

and σ_x is set as

$$\hat{\sigma}_x \leftarrow 0.2 * (2^S a) \quad \text{F-d}$$

Where a is a typical value of a voxel in the 3D reconstructed image, measured as

$$a = \sum_{i=1}^{M} \frac{b_i W_{ii} y_i}{\widetilde{M} N_c} \quad \text{F-e}$$

where N_c is the number of pixels along the column dimension of the detector and b_i is a binary indicator variable which is one in the non-zero parts of the y_i and $\widetilde{M}$ is the number of non-zero entries. These automated techniques while empirical have been observed to provide a high quality reconstruction (visually) across a range of conditions (noise levels, number of projections)[2] which makes it convenient because the user does not have to change the values in a real-time system as more data is acquired. In all the results shown in this paper, the sharpness value is set to S=0 and the SNR value to 30 dB. We also limit the maximum number of iterations to 200 for the reconstructions (512×512×200, 600×600×160, and 400×400×250 pixels[3]). The rest of the parameters were set to the default values of the software. Finally, naïve implementations of MBIR can be slow and impractical for large volumetric reconstructions. However, the svMBIR has a fast implementation that uses multiple CPU cores while also requiring fewer iterations to converge making it practical to deploy for our data sets.

Figure F1 displays a representative slice of the Ni-Cu sample reconstructed using two algorithms: the super voxel Model Based Iterative Reconstruction (svMBIR), and the conventional filtered back projection (FBP) method. As shown in this figure, the svMBIR method can generate higher quality reconstructed slices with the same number of sparse projections. Using the same attenuation scale, artifacts are more pronounced in the FBP slice (a) than the svMBIR one (b). Moreover, svMBIR has added benefit of automatic choice of regularization parameters to obtain a reasonable image quality, which is a more robust reconstruction method for our HyperCT system.

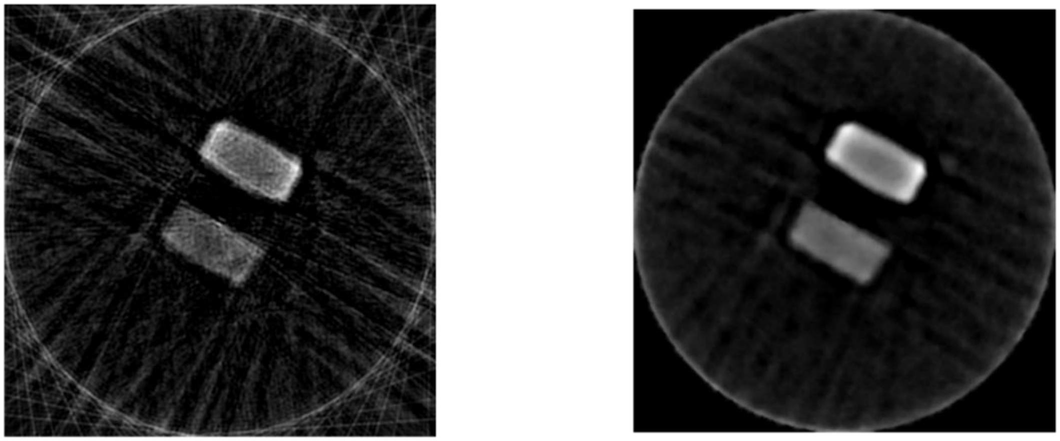

(a) (b)

Figure F1. Reconstructed slices of the Ni-Cu samples at $\lambda_2 = 4.18$ Å using (a) FBP and (b) svMBIR methods for Experiment I - 0° tilt. As seen in the figure, the svMBIR reconstruction yield better image quality and less artifacts when using the same number of sparse projections as for the FBP method.

References

1 Super-Voxel Model Based Ierative Reconstruction (SVMBIR) (2020).

2 Yang, D. *et al.* in *ICASSP 2023 - 2023 IEEE International Conference on Acoustics, Speech and Signal Processing (ICASSP).* 1-5.
