# Supplementary material for: A machine learning decision criterion for reducing scan time for hyperspectral neutron computed tomography systems
Source: Sci Rep. 2024 Jul 2;14:15171. doi: 10.1038/s41598-024-63931-x (PMC11220078; doi:10.1038/s41598-024-63931-x)
Supplement: Supplementary file 1 — Supplementary Information. [file 41598_2024_63931_MOESM1_ESM.zip › SREP-24-00554-s7.pdf]

## Appendix A

**Absolute and relative quality used for the design of the stopping criteria**

In this paper, we construct the reconstruction quality index (rQI) using two weighted elements: CNN based quality score and Averaged Change Index (ACI) between consecutive reconstructions (see Equation (6)). Here the CNN based quality score refers to the absolute quality of the reconstruction which is based on the quality features of reconstruction itself, such as contrast, sharpness, scale, etc., while the ACI aims to measure the improvement of the reconstruction after adding more projections.

In order to illustrate the motivation for using both ML and ACI, we have created a hypothetical scenario based on our existing sample. Instead of a uniform scanning strategy used at each new step, we assume there is an alternate sampling strategy (as in our recent work by Yang, D. et al. [55]) where a new orientation is proposed that is close to the existing angle because it can offer some quality benefits. Furthermore, we assume that we have not measured a large number of angles i.e. no matter which algorithm is used there are still artifacts in the current reconstruction. For such a case, the ACI values will be very large (almost no change in the overall reconstruction) but the absolute quality score will be very low – helping to avoid premature ending of the experiment.

We demonstrated one of the special cases with an NMC cathode sample. We generated the reconstructions *b, c* and *d* from 3 (at 40.47˚, 151.72˚, 82.96˚), 4 (at 40.47˚, 151.72˚, 82.96˚, 14.21˚) and 5 projections (at 40.47˚, 151.72˚, 82.96˚, 14.21˚, 81.5˚) and displayed a reconstructed slice in Figure A-1. Specially, one of the orientations for reconstruction *d* is very close to existed scanning angles. We calculated the ACI and set $\gamma$ as 0 which can isolate the ACI from the machine learning quality score (shown in Figure A-2). We find the ACI increased dramatically from 2 to 4. This is caused by the fact that the very similar new projection did not improve reconstruction a lot, which means the change in reconstruction quality is small and the ACI will be large. We also calculate the rQI after we add CNN scores ($\alpha = 0.6$). The averaged rQI change is slightly reduced by the CNN score. In this case, the reconstruction quality is obviously not sufficient to end the experiment, but the ACI may have reached a threshold. This is the reason why we need an independent metric to evaluate the reconstruction quality itself.

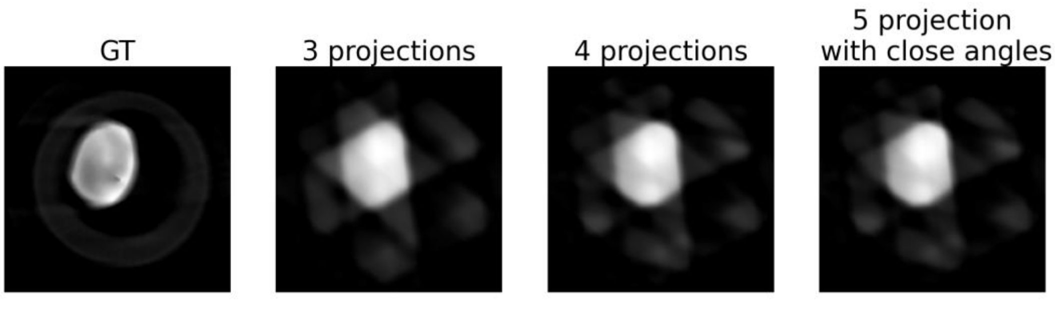


*Figure A-1 Reconstructed slices of the NMC battery sample using different numbers of projections (NMC cathode sample). From left to right: ground truth, 3 projections (reconstruction b), 4 projections (reconstruction c), and 5 projections (reconstruction d).*

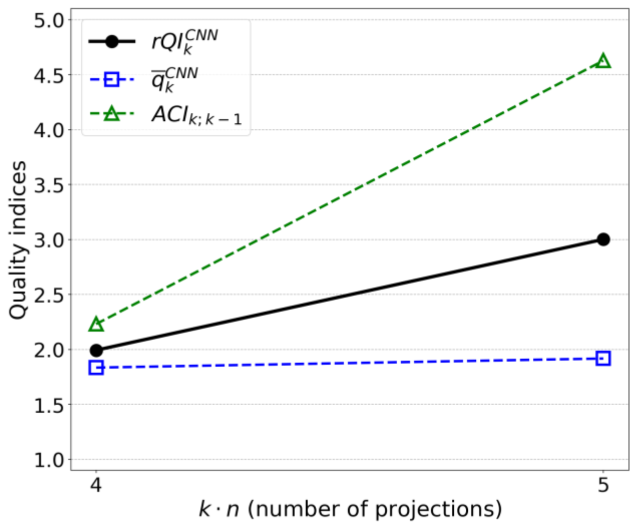


*Figure A-2 rQI plots along the projection number.*

We also introduced a user-defined parameter, α, to enable the design of a flexible stopping criterion which can weight the output of the reference-free CNN based on the reconstruction quality relative to the change in the subsequent reconstructions (ACI). If we have access to only limited amounts of training data (as can happen in neutron CT), the CNN score may not generalize across all new samples that are scanned at the instrument. In such cases, the beamline scientist who is setting up the experiment can manually adjust the value of α to be lower (to allow more projection measurements).

To illustrate this point and the impact of having α, we simulated a CT data set from the extended cardiac-torso (XCAT) phantom of size (256×256×256 pixels$^3$) (show in Figure A-3 (a)). The phantom's shape, structure, scale etc. are much more different from training data we used for the CNN model in this manuscript. We run the simulated experiment (mentioned in the main manuscript) on this phantom which means we simulate the forward sinogram using the golden-ratio angles and generate reconstructions every 3 projections (shown in Figure A-3 (b)-(d)). We calculate the CNN based scores ($\overline{q}_k$), $ACI_k$ and $rQI_k$ every 3 projections (plotted in Figure A-4). The ($\overline{q}_k$) score alone is a misleading stopping criterion in this case, i.e. it has a very high value even for this low-resolution reconstruction obtained from 6 projections. If we set the stopping threshold to 4.3, the rQI will reach the threshold only after 20 projections when α = 0.6. If we reduce the α value to 0.1, the influence of the CNN based score is decreased, the rQI reaches a threshold after 30 projections. The specific values of α are still empirical. In our experience, a value on the order of 0.6 is acceptable when there is a reasonable match between the new sample and the samples used during the CNN training.

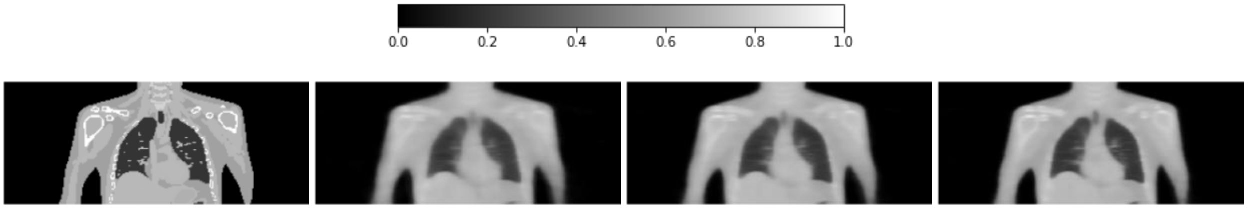


*(a) Ground Truth* | *(b) Reconstruction from 15 projection* | *(c) Reconstruction from 20 projection* | *(d) Reconstruction from 30 projection*

*Figure A-3 Vertically reconstructed slice of the medical phantom: ground-truth reconstruction (a), reconstruction from 15 (b), 20 (c) and 30 (d) projections, respectively.*

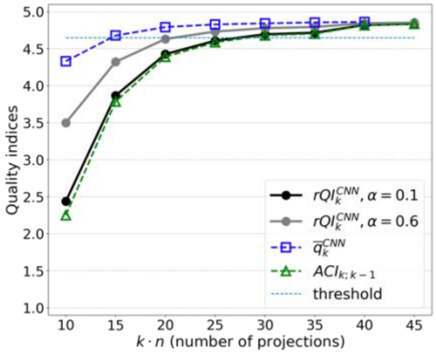


*Figure A-4 rQIs, CNN scores ($q_k^{CNN}$) and ACI as a function of the number of projections for a simulated experiment using an XCAT phantom.*
