# Supplementary figures and images for: A machine learning decision criterion for reducing scan time for hyperspectral neutron computed tomography systems
Source: Sci Rep. 2024 Jul 2;14:15171. doi: 10.1038/s41598-024-63931-x (PMC11220078; doi:10.1038/s41598-024-63931-x)

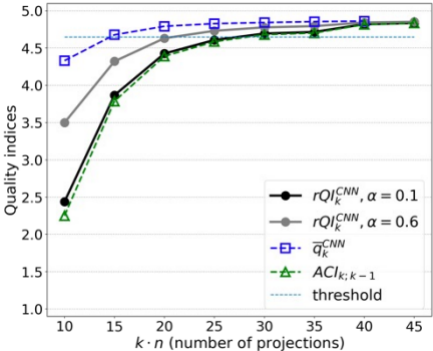

Supplement: Supplementary file 1 — Supplementary Information. [file 41598_2024_63931_MOESM1_ESM.zip › SREP-24-00554-s11.pdf]

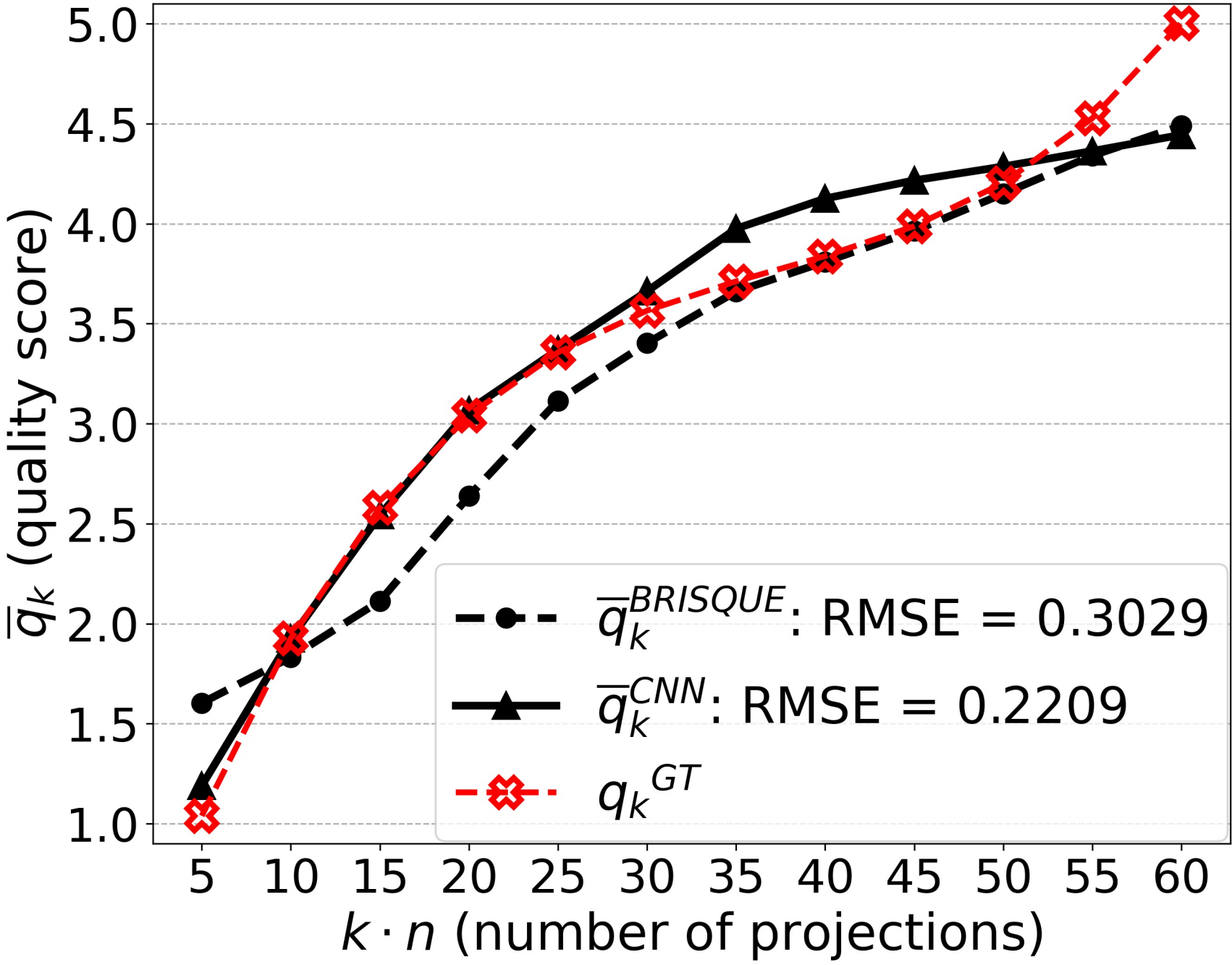

Supplement: Supplementary file 1 — Supplementary Information. [file 41598_2024_63931_MOESM1_ESM.zip › SREP-24-00554-s15.pdf]

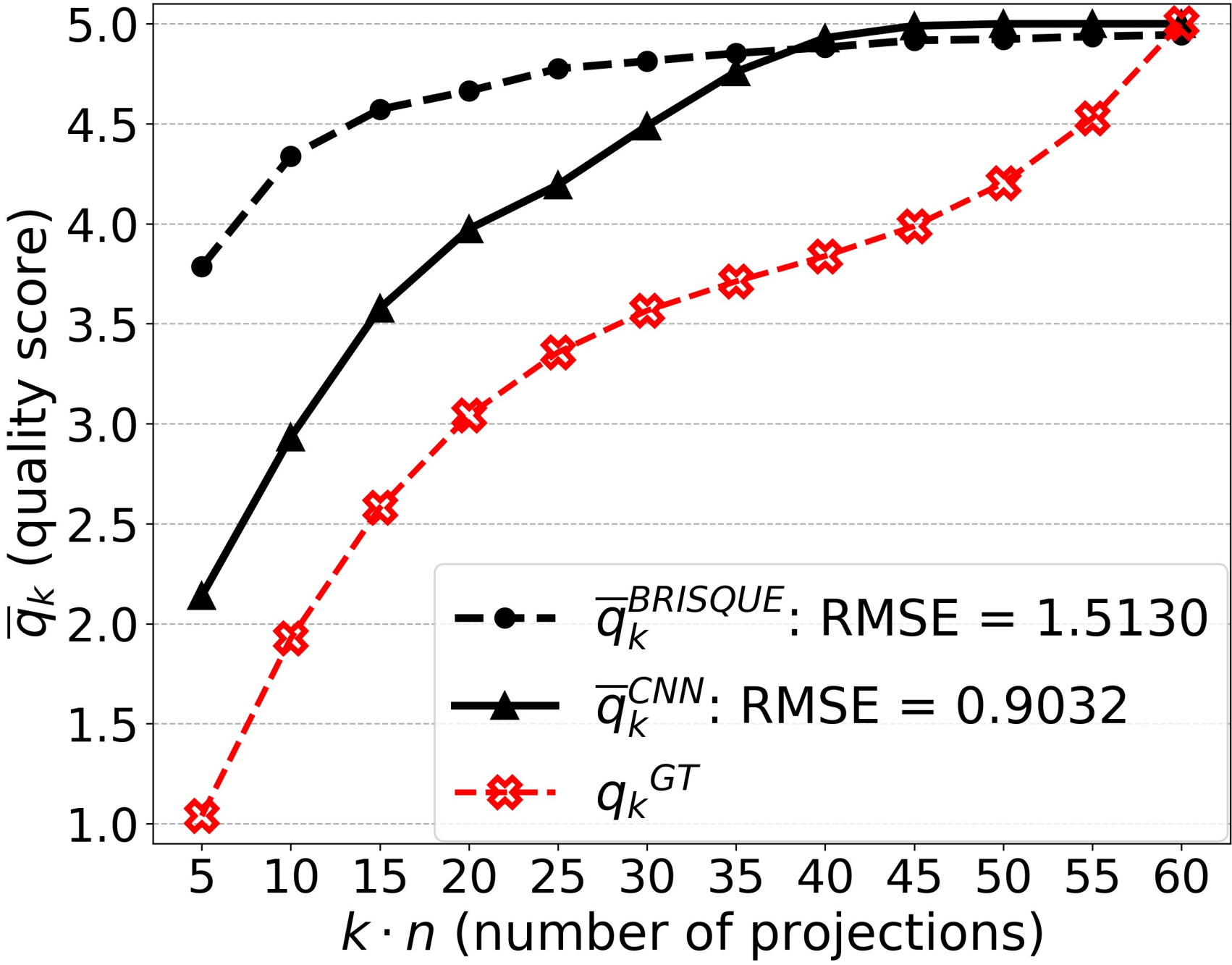

Supplement: Supplementary file 1 — Supplementary Information. [file 41598_2024_63931_MOESM1_ESM.zip › SREP-24-00554-s16.pdf]

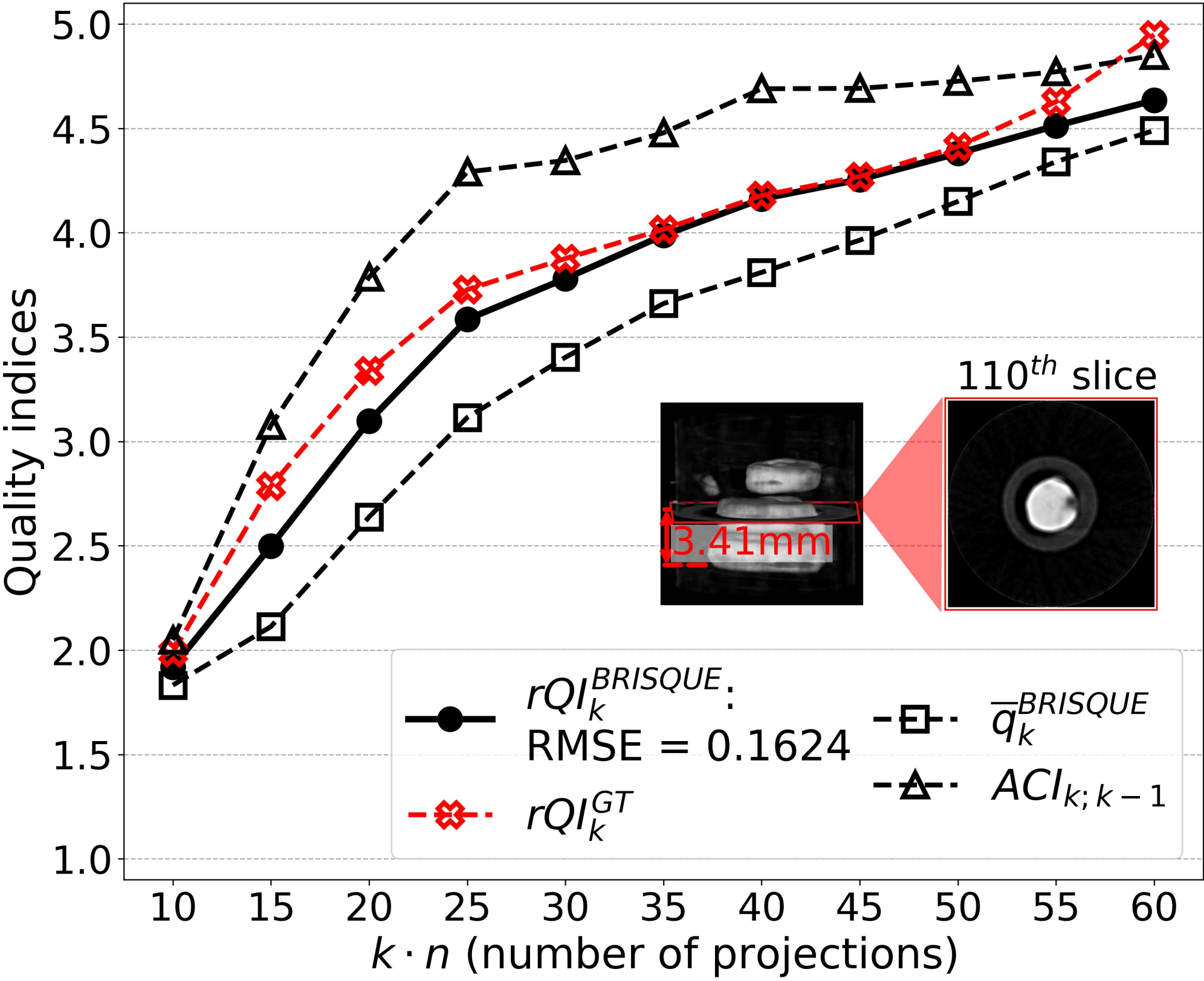

Supplement: Supplementary file 1 — Supplementary Information. [file 41598_2024_63931_MOESM1_ESM.zip › SREP-24-00554-s17.pdf]

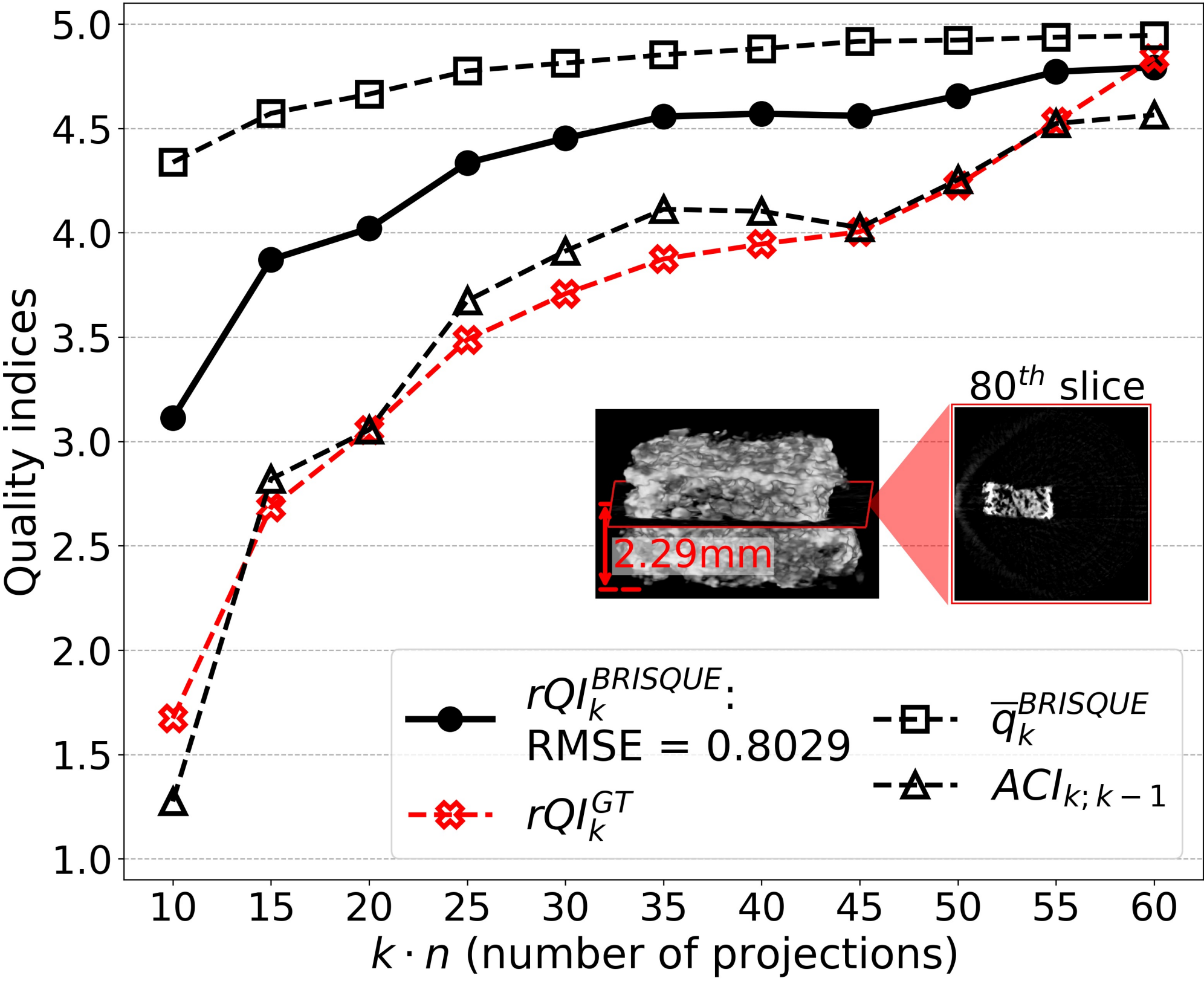

Supplement: Supplementary file 1 — Supplementary Information. [file 41598_2024_63931_MOESM1_ESM.zip › SREP-24-00554-s18.pdf]

Number of voxels

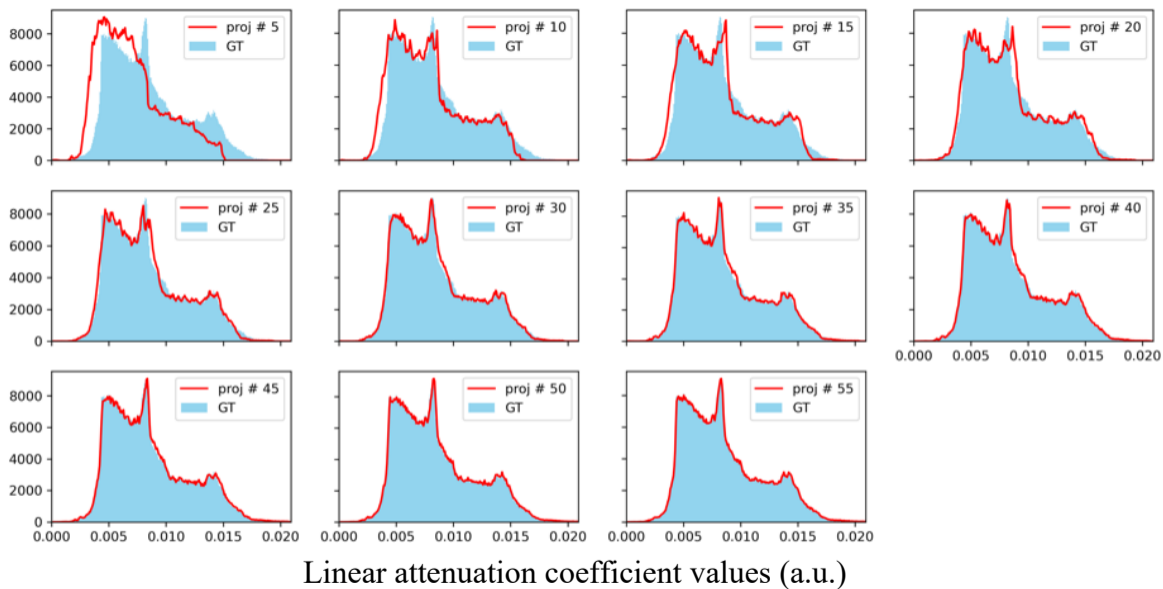

Supplement: Supplementary file 1 — Supplementary Information. [file 41598_2024_63931_MOESM1_ESM.zip › SREP-24-00554-s20.pdf]

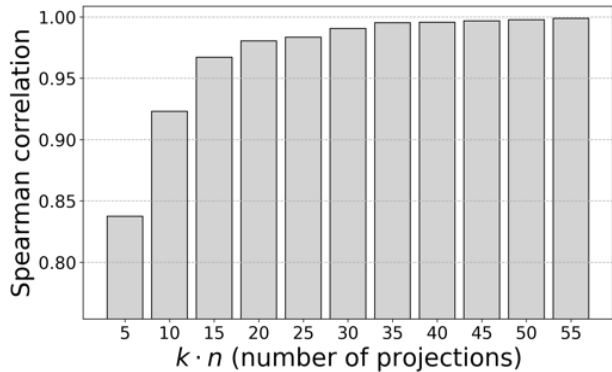

(a)

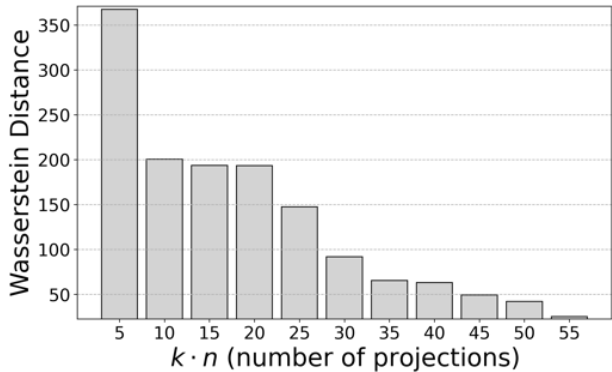

(b)

Supplement: Supplementary file 1 — Supplementary Information. [file 41598_2024_63931_MOESM1_ESM.zip › SREP-24-00554-s21.pdf]

Number of voxels

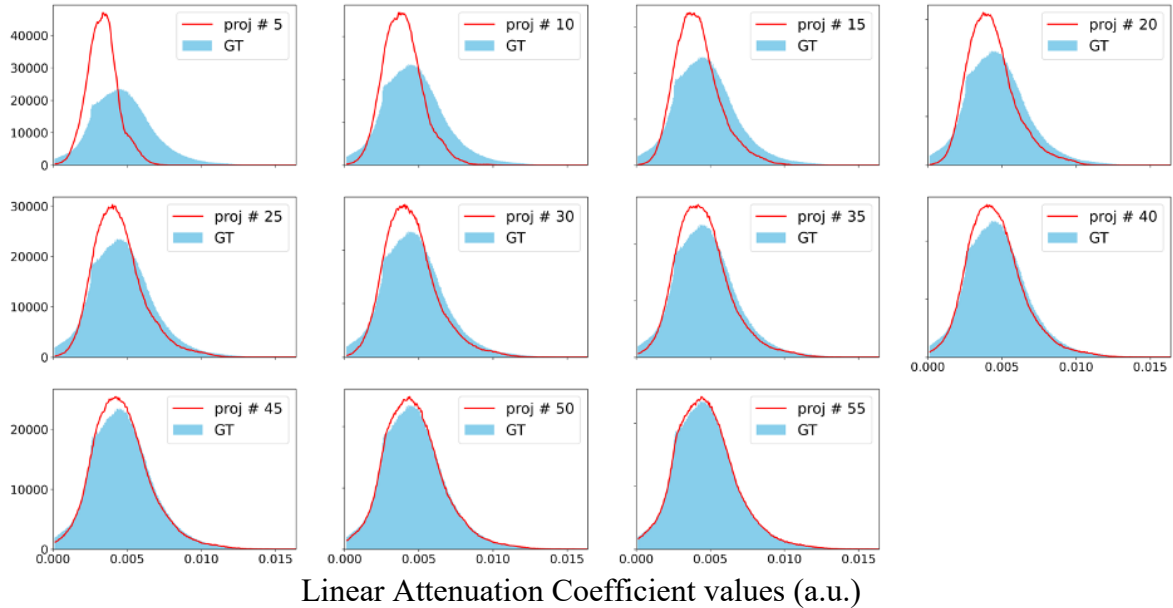

Supplement: Supplementary file 1 — Supplementary Information. [file 41598_2024_63931_MOESM1_ESM.zip › SREP-24-00554-s22.pdf]

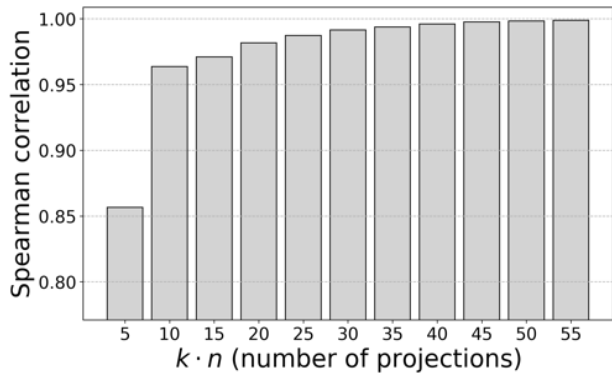

(a)

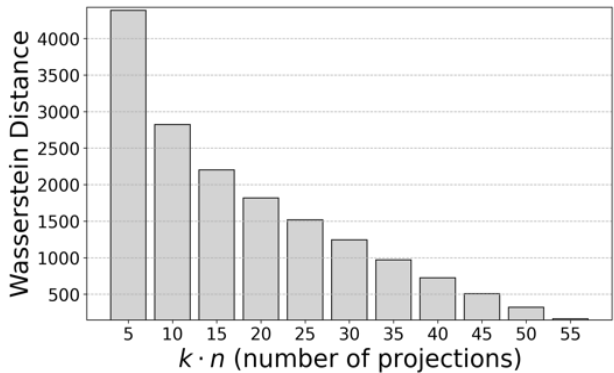

(b)

Supplement: Supplementary file 1 — Supplementary Information. [file 41598_2024_63931_MOESM1_ESM.zip › SREP-24-00554-s23.pdf]

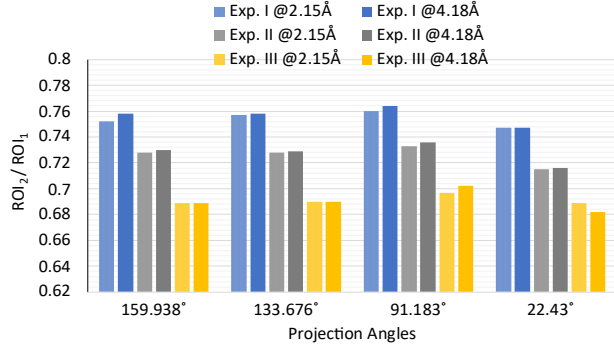

(a)

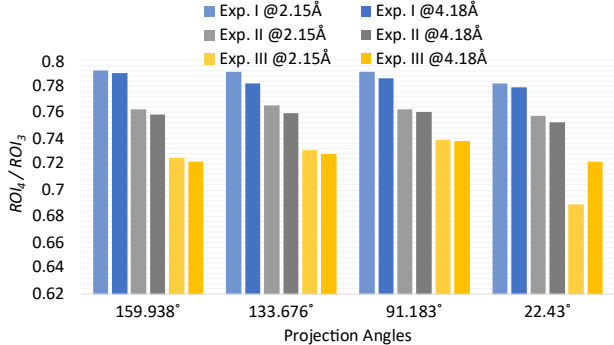

(b)

Supplement: Supplementary file 1 — Supplementary Information. [file 41598_2024_63931_MOESM1_ESM.zip › SREP-24-00554-s26.pdf]

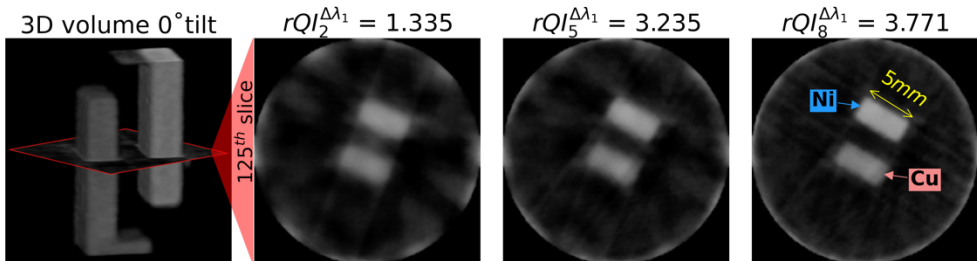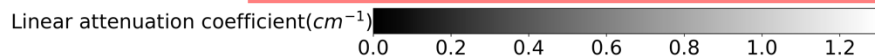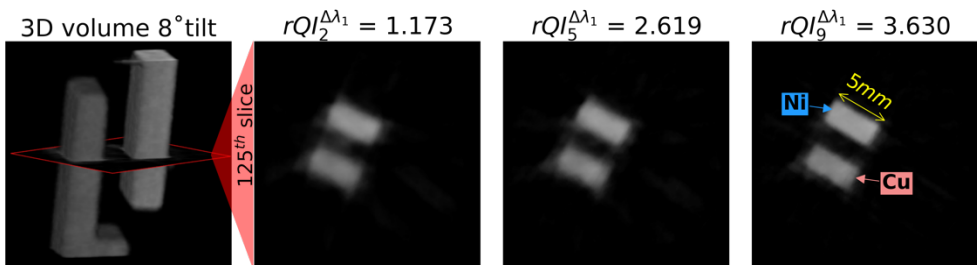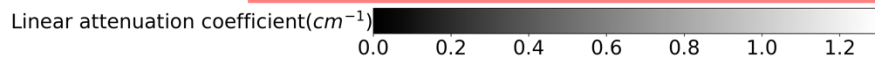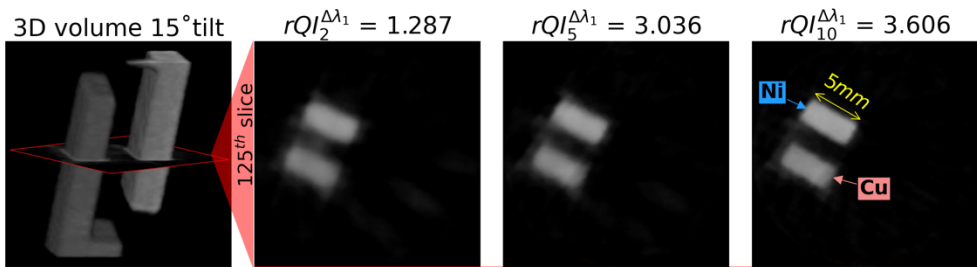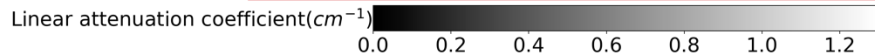

Supplement: Supplementary file 1 — Supplementary Information. [file 41598_2024_63931_MOESM1_ESM.zip › SREP-24-00554-s27.pdf]

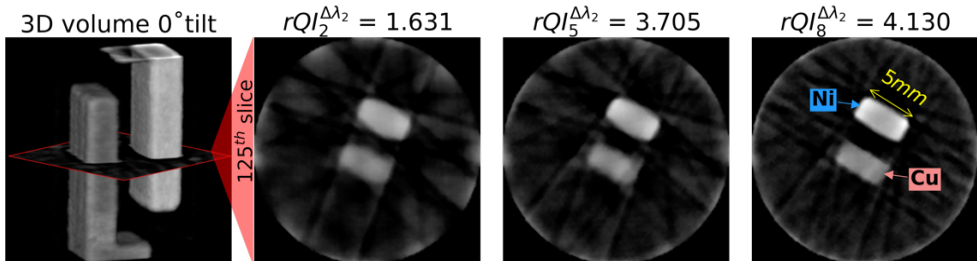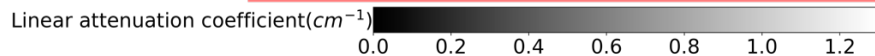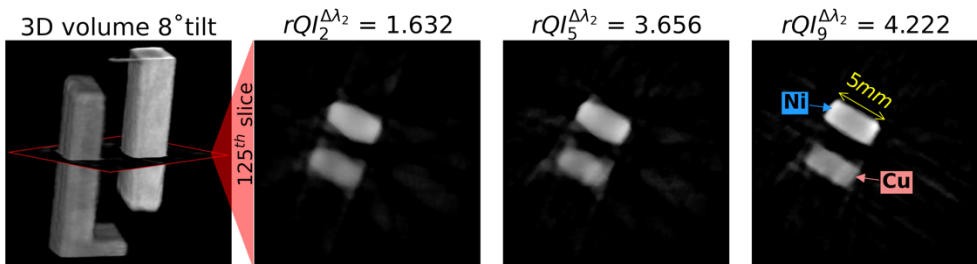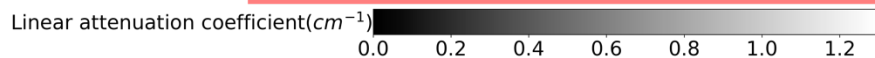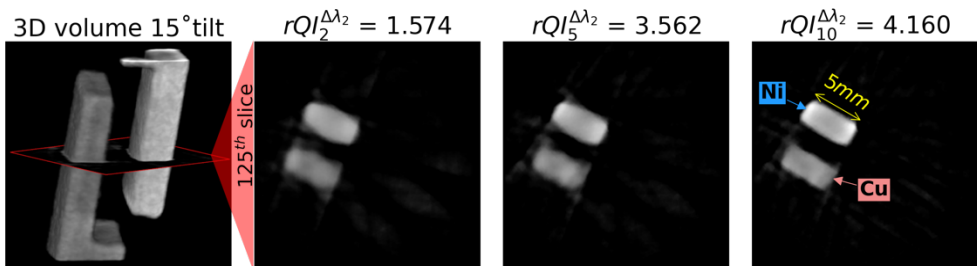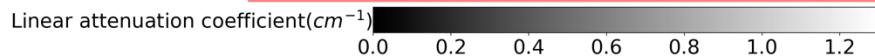

Supplement: Supplementary file 1 — Supplementary Information. [file 41598_2024_63931_MOESM1_ESM.zip › SREP-24-00554-s28.pdf]

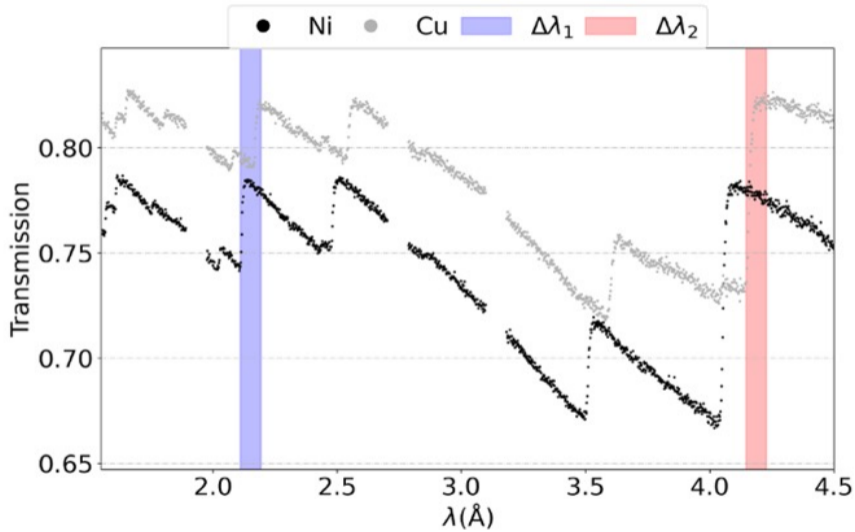

Supplement: Supplementary file 1 — Supplementary Information. [file 41598_2024_63931_MOESM1_ESM.zip › SREP-24-00554-s29.pdf]

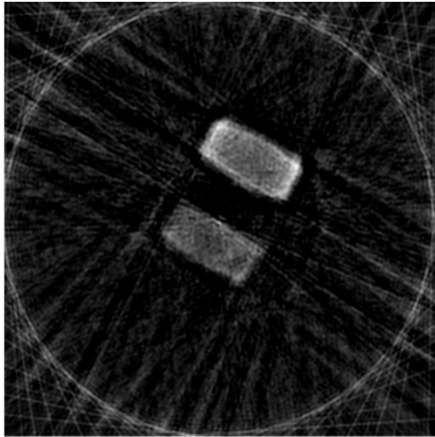

(a)

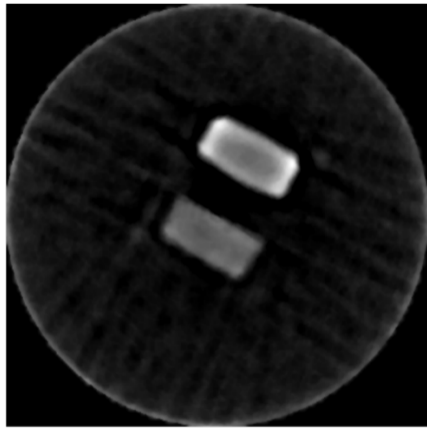

(b)

Supplement: Supplementary file 1 — Supplementary Information. [file 41598_2024_63931_MOESM1_ESM.zip › SREP-24-00554-s31.pdf]

GT

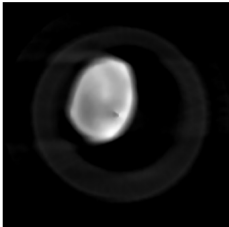

3 projections

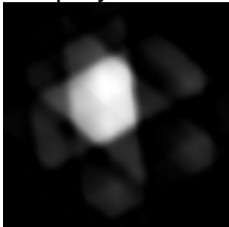

4 projections

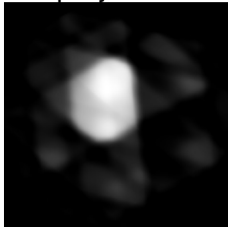

5 projection  
with close angles

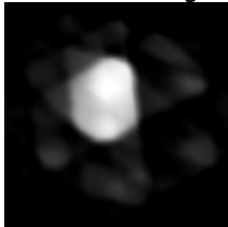

Supplement: Supplementary file 1 — Supplementary Information. [file 41598_2024_63931_MOESM1_ESM.zip › SREP-24-00554-s8.pdf]

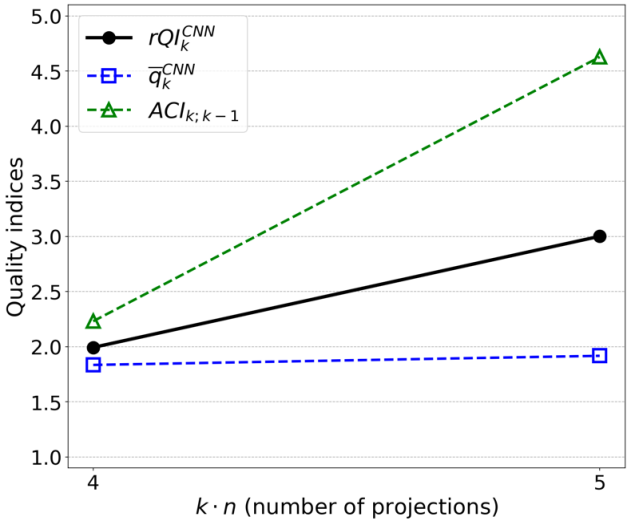

Supplement: Supplementary file 1 — Supplementary Information. [file 41598_2024_63931_MOESM1_ESM.zip › SREP-24-00554-s9.pdf]
